# Long-term effects of SARS-CoV-2 infection on blood vessels and blood pressure – LOCHINVAR

**DOI:** 10.1097/HJH.0000000000004013

**Published:** 2025-04-10

**Authors:** Stefanie Lip, Tran Q.B. Tran, Rebecca Hanna, Sarah Nichol, Tomasz J. Guzik, Christian Delles, John McClure, Linsay McCallum, Rhian M. Touyz, Colin Berry, Sandosh Padmanabhan

**Affiliations:** a School of Cardiovascular and Metabolic Health, University of Glasgow; b Queen Elizabeth University Hospital, Glasgow; c Centre for Cardiovascular Science, University of Edinburgh, Edinburgh, Scotland, UK; d Research Institute of the McGill University Health Centre, McGill University, Montreal, Canada

**Keywords:** 6-min walk test, blood pressure, brachial flow mediated dilatation, COVID-19, EQ-5D-3L, hypertension, quality of life, renin–angiotensin–aldosterone system, SARS-CoV-2

## Abstract

**Objective:**

The COVID-19 pandemic has been linked to endothelial dysfunction and renin–angiotensin–aldosterone system (RAAS) dysregulation, potentially worsening hypertension. Longitudinal studies are needed to establish COVID-19's lasting effects on blood pressure (BP) and endothelial function. Our objective was to determine whether COVID-19 increases future hypertension risk by comparing BP and endothelial function in nonhypertensive COVID-19 survivors with nonhypertensive controls.

**Methods:**

This single-centre prospective longitudinal study included participants without hypertension history, with cases being hospital-admitted COVID-19 survivors and controls having negative SARS-CoV-2 antibody tests. Ambulatory blood pressure monitoring, flow-mediated dilatation (FMD), 6-min walk test (6MWT), and quality of life (QoL) assessments were conducted at baseline and 12 months. RAAS phenotyping was performed at baseline. Data analysis used paired t-tests and multivariable regression on full and per-protocol datasets.

**Results:**

The full (*n* = 97) and per-protocol (*n* = 66) datasets included 37 and 15 cases respectively. Median ages (IQR: interquartile range) were 49.0 (43.0–53.5) and 50.0 (42–54.0) years. Baseline RAAS parameters were similar. Multivariable adjusted analyses in the per-protocol group showed SARS-CoV-2 positive participants had a 12-month increase in mean systolic BP (4.57 mmHg, [95% CI –0.04 to 9.18], *P* = 0.052), diastolic BP (4.46 mmHg [1.01 to 7.90], *P* = 0.012), decrease in FMD (–3.15% [–6.33 to 0.04], *P* = 0.053) and improvement in 6MWT (145.6 m [49.1 to 242.1], *P* = 0.004) compared to controls. QoL assessments indicated continued challenges for recovered COVID-19 individuals at 12 months.

**Conclusions:**

Persistent vascular dysfunction and BP increase post-COVID-19 underscore the need for further studies on the long-term risk of hypertension and cardiovascular disease.

**Clinical trial registration:**

https://clinicaltrials.gov/study/NCT05087290

## Graphical abstract

**Figure:**
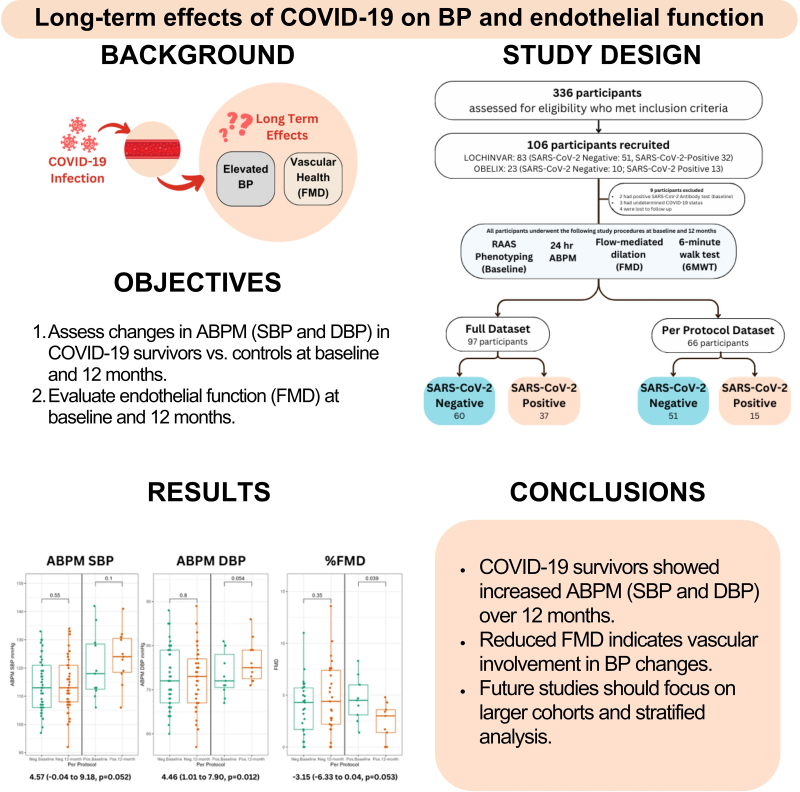

## INTRODUCTION

The coronavirus disease 2019 (COVID-19) pandemic, caused by the severe acute respiratory syndrome coronavirus 2 (SARS-CoV-2), has had unprecedented global health, economic, and societal impacts. Initially characterised as a respiratory illness, COVID-19 has since been recognised to have significant multiorgan effects, with the cardiovascular system being particularly affected. While acute cardiovascular complications of COVID-19, such as myocarditis, arrhythmias, and thromboembolic events, have been well documented, there is a growing body of evidence suggesting that the virus can also have long-term cardiovascular consequences including hypertension [1–3]. Individuals who have recovered from COVID-19 may exhibit persistent endothelial dysfunction and elevated blood pressure (BP). In COVID-19 survivors, Gao *et al.* found that endothelial dysfunction, as indicated by reduced brachial flow mediated dilatation (FMD), persisted for up to 327 days postinfection and Faria *et al.* found greater carotid-femoral pulse wave velocity and lower FMD at 3 months post-COVID [4,5]. This aligns with findings from Serviente *et al.*, who reported long-term inflammation and oxidative stress contributing to endothelial impairment in this population [6]. Endothelial dysfunction is a critical precursor to atherosclerosis and cardiovascular diseases resulting from mechanisms that disrupt its normal role in vascular homeostasis by regulating blood vessel tone, blood flow, and the balance between coagulation and fibrinolysis. Endothelial dysfunction is characterised by a diminished capacity for endothelium-dependent vasodilation, commonly assessed clinically using FMD. This dysfunction leads to increased vascular resistance and hypertension, setting the stage for adverse cardiovascular events. Conversely, hypertension can worsen endothelial dysfunction indirectly that can increase cardiovascular risk. The renin–angiotensin–aldosterone system (RAAS) is another key regulator of BP and fluid balance. SARS-CoV-2 interacts with the RAAS by binding to the angiotensin-converting enzyme 2 (ACE2) receptor, which is expressed in various tissues, including the lungs, heart, kidneys, and blood vessels. The downregulation of ACE2 due to viral entry can disrupt the RAAS balance, potentially leading to elevated levels of angiotensin II thereby contributing to hypertension and endothelial injury [7].

Recent studies examining the relationship between COVID-19 and hypertension have yielded mixed results, largely due to the limitations of retrospective and observational designs, including small sample sizes, lack of control groups, and short follow-up durations, which make it challenging to draw definitive conclusions. While some studies report elevated blood pressure (BP) shortly after infection, others show no significant BP increase over longer follow-up periods. Furthermore, studies assessing endothelial function via flow-mediated dilation (FMD) in COVID-19 patients are limited by small sample sizes. A key gap in current knowledge is whether the impact of SARS-CoV-2 on endothelial function is progressive, potentially leading to sustained increases in BP over time.

The primary objective of this study is to investigate the long-term effects of COVID-19 on BP and endothelial function. Specifically, the study aims to: assess changes in 24-h ambulatory BP monitoring (ABPM) systolic and diastolic BP, in COVID-19 survivors compared to controls; evaluate endothelial function using FMD of the brachial artery at baseline and after 12 months. This study is based on the hypothesis that endothelial dysfunction associated with COVID-19 persists well beyond recovery, and this will be detectable through longitudinal evaluations of ABPM and FMD. In contrast, these measurements are not expected to be substantially affected in contemporaneous control subjects without a history of COVID-19.

Our study presents several strengths compared to existing literature. We provide data at two separate time points (baseline and 12 months) and use of 24-h ABPM which is regarded as the reference method for diagnosing hypertension. In addition, 24-h ABPM is an objective measure and not susceptible to assessor or participant bias. Importantly, our study groups (SARS-CoV-2 negative and positive) did not have a prior history of hypertension or treatment with BP lowering drugs, limiting reducing potential confounding and allowing for tracking of longitudinal BP changes potentially attributable to COVID-19 infection. Additionally, %FMD was measured longitudinally as a marker of endothelial dysfunction providing valuable insights into vascular health in recovered COVID-19 individuals.

## METHODS

### Study design

This is a single-centre prospective (Glasgow, UK), longitudinal observational study.

### Study population and recruitment

**FIGURE 1:**
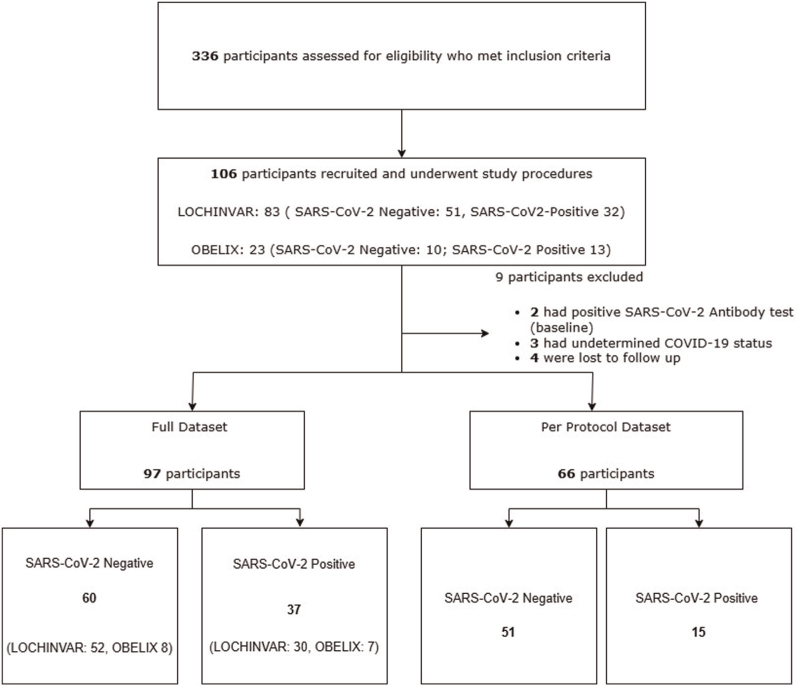
Study flow diagram.

Patients admitted to the Queen Elizabeth University Hospital (QEUH) initial assessment and acute receiving units (1 September 2020–31 December 2021) who presented with COVID-19 or non-COVID-19 illness were recruited to the study if they met inclusion and exclusion criteria. The first patient's study visit was the 17th of November 2021. The main inclusion criteria were participants aged between 30 and 60 years of age, with no history of hypertension and not on antihypertensive medications. Further details on study screening, study recruitment, inclusion and exclusion criteria have been explained in the protocol paper [8]. Participants who previously took part in the cross-sectional COVID-19 Blood Pressure Endothelium Interaction Study (OBELIX) study who had consented to be re-contacted were invited to take part in the 12-month follow-up visits in the LOCHINVAR study. Definitions of the SARS-CoV-2 positive and negative group details on clinical data collection, ethics and approvals can be found in Supplementary Methods. The study flow diagram is shown in Figure 1.

### Study procedures

The study procedures have been previously described [8]. At the baseline visit, routine blood samples (haematology and biochemistry) and RAAS fingerprinting were obtained. BP measurements including office BP (study visit) and ABPM (Spacelabs 90217RM) were recorded. FMD was performed using UNEX EF38G device (UNEX Corporation, Nagoya, Japan), with %FMD collected. 6-Minute Walk Test (6MWT) was conducted to assess exercise tolerance. Participants reported quality of life outcomes by completing the EuroQol five-dimension three-level (EQ-5D-3L) questionnaire including the EuroQol Visual Analogue Scale (EQ-VAS). All participants were invited to attend a follow up visit at 12 months where all study procedures were conducted except RAAS fingerprinting.

### Study outcomes

#### Primary outcome

The primary outcome is the change in average 24-h ABPM SBP at 12 months from baseline in the SARS-CoV-2 positive and SARS-CoV-2 negative groups.

#### Secondary outcomes

Secondary outcomes include 24-h ABPM diastolic BP (DBP); Day ABPM Systolic BP (SBP); Day ABPM DBP; Night ABPM SBP; Night ABPM DBP; % FMD; 6MWT, EQ-5D-3L Dimensions, EQ-5D-3L Index, EQ-5D-VAS and 24-h Urine Sodium.

### Statistical methods and statistical analysis

The sample size calculation has been previously described [8]. Group characteristics of continuous variables are described as mean ± standard deviation (SD) or median and IQR based on their distributions. Categorical data are summarised as frequencies (and percentages). Nonnormally distributed variables (renin, NT-pro-BNP, Ang II [1–8], Ang [1–7], Ang I [1–10], Ang [1–5]) were log transformed for analysis. Aldosterone was dichotomised into a binary variable split at the local NHS laboratory. We used independent two-sample Welch t-tests or chi-square tests of association for group comparisons where appropriate. We tested hypotheses using univariable and multivariable adjusted linear regression to explore the association between COVID-19 status and outcomes. We analysed longitudinal changes in ABPM, FMD, and 6MWT, by including the corresponding baseline values in the regression models in addition to other covariates. The Kruskal-Wallis test was used to compare the two groups at baseline for EQ-5D-3L without assuming normality. Wilcoxon signed-rank test was utilised to compare within subject changes for both timepoints for EQ-5D-3L Index and VAS scores and multivariate regression models with adjustment for confounders were used on the change of both parameters over 12 months. For the primary outcomes, the p-value for significance was set at 0.05. For the secondary outcomes, the nominal p-value threshold of *P* < 0.05 and the Bonferroni adjusted *P* < 0.0055 given the multiple tests carried out are presented. During the study, the evolution of SARS-CoV-2 virus, the roll-out of vaccines and antiviral therapies resulted in changes in public health advice and public risk perception. This resulted in challenges in recruiting participants for the study and contributed heterogeneity in the study population mainly related to timing of the baseline visit which exceeded 28 weeks in many cases. To address this, analyses were carried out in the full data set and per protocol data set (baseline visit time frame of 41–200 days post COVID-19 infection and baseline visits after year 2020). All analyses were conducted using R Software version 4.3.3.

## RESULTS

The results of the per-protocol analyses are presented here, with the results of the full dataset presented in Supplementary Tables (Tables S1–S8, Supplementary Digital Content) and Supplementary Figures S1–11, Supplementary Digital Content).

### Baseline characteristics

The study enrolled 97 participants who comprised the full dataset, of whom 66 were included in the per-protocol dataset (Fig. 1 and Table 1). Both datasets exhibited similar demographic and laboratory features. Participants in the SARS-CoV-2 positive and negative groups were comparable in age, median 49.0 (IQR 43.0–53.5) years versus 50.0 (42.0–54.0), respectively. Most participants were female, white Caucasian, had no history of diabetes, and consumed 1–14 units of alcohol per week. There was no significant difference in body mass index (BMI) between the groups. Baseline BP (office and ambulatory) and %FMD were comparable between groups. Significant baseline differences between groups were observed in the gender of participants, serum sodium, urea, creatinine, and HbA1c levels (Table S1, Supplementary Digital Content).

**TABLE 1:** Baseline characteristics per-protocol and full dataset (SARS-CoV-2 negative group vs. SARS-CoV-2 positive group)

		Full dataset (*n* = 97)			Per-protocol dataset (*n* = 66)		
Label (baseline)	Levels	SARS-CoV-2 Neg (*n* = 60)	SARS-CoV-2 Pos (*n* = 37)	*P*	SARS-CoV-2 Neg (*n* = 51)	SARS-CoV-2 Pos (*n* = 15)	*P*
Age (years)	Median (IQR)	49.5 (41.0–54.0)	48.0 (44.0–54.0)	0.680	50.0 (42.0–54.0)	49.0 (43.0–53.5)	0.939
Sex (*n*, %)	Female	48 (80.0)	19 (51.4)	0.006	42 (82.4)	6 (40.0)	**0.003**
	Male	12 (20.0)	18 (48.6)		9 (17.6)	9 (60.0)	
Office SBP (mmHg)	Mean (SD)	122.1 (12.8)	123.6 (12.9)	0.594	122.6 (13.2)	122.8 (13.1)	0.945
Office DBP (mmHg)	Mean (SD)	75.4 (10.1)	79.3 (9.6)	0.066	74.8 (10.4)	78.7 (5.6)	0.169
ABPM SBP (mmHg)	Mean (SD)	114.9 (9.8)	119.4 (12.5)	0.052	114.7 (10.0)	117.8 (12.3)	0.327
ABPM DBP (mmHg)	Mean (SD)	73.1 (6.4)	75.1 (7.3)	0.148	73.3 (6.7)	72.9 (4.6)	0.827
ABPM SBP (day) (mmHg)	Mean (SD)	117.9 (10.1)	121.5 (13.0)	0.127	117.6 (10.3)	121.9 (12.0)	0.177
ABPM DBP (day) (mmHg)	Mean (SD)	75.6 (6.9)	76.6 (8.4)	0.514	75.5 (7.2)	76.1 (4.5)	0.760
ABPM SBP (night) (mmHg)	Mean (SD)	104.7 (10.1)	108.6 (14.2)	0.121	104.7 (10.1)	105.3 (14.0)	0.862
ABPM DBP (night) (mmHg)	Mean (SD)	64.6 (6.0)	66.5 (9.4)	0.223	64.9 (6.1)	62.2 (5.8)	0.137
Na (mmol/l)	Mean (SD)	138.8 (1.8)	140.2 (1.6)	<0.001	138.6 (1.8)	140.4 (1.7)	**0.001**
Urea (mmol/l)	Median (IQR)	3.9 (3.4–4.6)	4.7 (4.2–5.4)	0.003	3.9 (3.5–4.7)	4.9 (4.3–5.5)	**0.011**
Creatinine (μmol/l)	Mean (SD)	65.2 (13.0)	73.3 (17.0)	0.009	65.2 (11.7)	75.7 (18.7)	**0.010**
HbA1c (mmol/mol)	Mean (SD)	34.5 (2.6)	37.1 (5.5)	0.003	34.3 (2.6)	37.1 (2.9)	**0.001**
%FMD	Median (IQR)	4.7 (2.0–8.5)	4.5 (2.4–6.5)	0.461	4.7 (2.0–8.5)	4.6 (2.6–6.8)	0.662
6MWT distance (m)	Median (IQR)	654.0 (570.0–750.0)	624.0 (544.5–727.5)	0.656	654.0 (570.0–750.0)	642.0 (534.0–747.0)	0.800

This table presents the baseline demographics and clinical characteristics of the participants in the full dataset and per-protocol dataset stratified by SARS-CoV-2 status (positive or negative). Continuous variables are reported as mean (standard deviation), and categorical variables are presented as frequency (percentage). P-values indicate group differences assessed using independent t-tests for continuous variables and chi-square tests for categorical variables. %FMD, percentage change in brachial flow mediated dilatation; 6MWT, 6-minute walk test; DBP, diastolic blood pressure; HbA1c, serum glycosylated haemoglobin; Na, serum sodium; SBP, systolic blood pressure.

### Follow-up characteristics

At 12 months ABPM SBP (SARS-CoV-2 positive group: 127.5 ± 10.4) mmHg vs. SARS-CoV-2 negative Group: 114.2 ± 9.5 mmHg, *P* = 0.004) showed significant differences including day and night ABPM SBP. (Table 2). The SARS-CoV-2 positive group had a significantly lower %FMD [median 1.8 (IQR 1.4 to 3.5) vs. 4.6 (2.6 to 7.0)].

**TABLE 2:** 12-Month characteristics for full and per-protocol data set (SARS-CoV-2 negative group vs. SARS-CoV-2 positive group)

		Full dataset (*n* = 97)			Per-protocol dataset (*n* = 66)		
Label (12 months)	Levels	SARS-CoV-2 Neg *n* = 60	SARS-CoV-2 Pos *n* = 37	*P*	SARS-CoV-2 Neg *n* = 51	SARS-CoV-2 Pos *n* = 15	*P*
Age	Median (IQR)	49.5 (41.0–54.0)	48.0 (44.0–54.0)	0.680	50.0 (42.0–54.0)	49.0 (43.0–53.5)	0.939
Sex (*n*, %)	Female	48 (80.0)	19 (51.4)	0.006	42 (82.4)	6 (40.0)	**0.003**
	Male	12 (20.0)	18 (48.6)		9 (17.6)	9 (60.0)	
Office SBP (mmHg)	Mean (SD)	119.9 (12.8)	127.8 (14.6)	0.017	119.5 (13.2)	128.0 (15.0)	0.051
Office DBP (mmHg)	Mean (SD)	76.7 (8.9)	80.5 (8.3)	0.074	76.3 (8.9)	81.2 (6.2)	0.069
ABPM SBP (mmHg)	Mean (SD)	114.7 (9.5)	121.6 (10.8)	0.007	114.2 (9.5)	123.9 (9.6)	**0.004**
ABPM DBP (mmHg)	Mean (SD)	72.4 (6.8)	75.3 (5.6)	0.072	72.4 (6.9)	76.3 (4.7)	0.085
ABPM SBP (day) (mmHg)	Mean (SD)	117.2 (10.4)	124.5 (11.2)	0.008	116.9 (10.3)	127.5 (10.4)	**0.004**
ABPM DBP (day) (mmHg)	Mean (SD)	74.2 (7.9)	77.6 (6.2)	0.071	74.5 (7.3)	78.7 (5.9)	0.077
ABPM SBP (night) (mmHg)	Mean (SD)	104.0 (10.6)	110.8 (10.7)	0.012	104.0 (10.7)	112.8 (9.3)	**0.015**
ABPM DBP (night) (mmHg)	Mean (SD)	64.7 (7.6)	66.5 (6.4)	0.332	65.0 (7.7)	67.5 (4.1)	0.304
Urea (mmol/l)	Median (IQR)	4.1 (3.3–4.8)	5.2 (4.1–5.5)	0.004	4.1 (3.3–4.8)	5.2 (4.2–5.4)	**0.041**
Creatinine (μmol/l)	Mean (SD)	67.2 (13.1)	77.8 (15.3)	0.002	67.7 (11.6)	80.0 (16.3)	**0.003**
HbA1C (mmol/mol)	Mean (SD)	35.6 (2.8)	37.5 (5.1)	0.043	35.5 (2.9)	37.5 (2.0)	**0.023**
%FMD	Median (IQR)	5.0 (2.6–7.4)	2.4 (1.1–4.0)	0.002	4.6 (2.6–7.0)	1.8 (1.4–3.5)	**0.009**
6MWT distance (m)	Median (IQR)	750.0 (672.0–912.0)	897.0 (816.0–1011.0)	0.015	768.0 (708.0–930.0)	900.0 (858.0–1002.0)	**0.022**

This table presents the 12-month demographics and clinical characteristics of the participants in the full dataset and per-protocol dataset stratified by SARS-CoV-2 status (positive or negative). Continuous variables are reported as mean (standard deviation), and categorical variables are presented as frequency (percentage). P-values indicate group differences assessed using independent t-tests for continuous variables and chi-square tests for categorical variables. %FMD, percentage change in brachial flow mediated dilatation; 6MWT, 6-minute walk test; ABPM, ambulatory blood pressure; DBP, diastolic blood pressure; HbA1c, serum glycosylated haemoglobin; IQR, interquartile range; Na, serum sodium; SBP, systolic blood pressure; SD, standard deviation.

### Ambulatory blood pressure measurement

**FIGURE 2:**
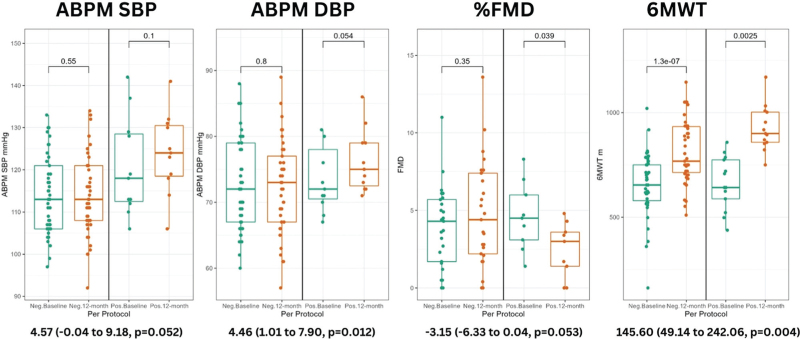
Paired ABPM SBP, ABPM DBP, % FMD, 6MWT (per-protocol dataset – SARS-CoV-2 negative group vs. SARS-CoV-2 positive group). This figure demonstrates the paired analysis of ABPM SBP, ABPM DBP, %FMD, and 6MWT for both SARS-CoV-2 positive (*n* = 15) and SARS-CoV-2 negative (*n* = 51) groups at baseline and 12 months. The numbers above the brackets are the *P*-values for each paired analyses at baseline and 12 months for each group. The *P*-value at the bottom is the multivariable regression analysis after adjusting for age, sex, and BMI.

The results of within group paired analyses of BP at baseline and 12 months are presented in Fig. 2. Multivariable adjusted analysis in the per protocol dataset are presented in Table 3 and show the baseline to 12-month change in ABPM DBP was significantly greater in the SARS-CoV-2 positive group compared to the SARS-CoV-2 negative group (4.46 mmHg 95% CI [1.01 to 7.90], *P* = 0.012) while the difference just missed statistical significance for ABPM SBP change (4.57 mmHg [–0.04 to 9.18], *P* = 0.052). The results were consistent in in magnitude and direction in the full dataset (Table S6, Supplementary Digital Content).

**TABLE 3:** Longitudinal Regression Analyses - Per-protocol (Coefficient SARS-COV-2 Positive Group vs SARS-COV-2 Negative Group) at 12 months

Per-protocol Dataset	Mean (SD)	Univariable model	Multivariable (age, sex, BMI, baseline measure)
Office SBP (mmHg)	128.0 (15.0)	8.50 (−0.02–17.02, *P* = 0.051)	5.13 (−1.10–11.37, *P* = 0.105)
Office DBP (mmHg)	81.2 (6.2)	4.88 (−0.40–10.16, *P* = 0.069)	0.61 (−3.22–4.44, *P* = 0.750)
ABPM SBP (mmHg)	123.9 (9.6)	9.71 (3.30–16.12, *P* = 0.004)	4.57 (−0.04–9.18, *P* = 0.052)
ABPM DBP (mmHg)	76.3 (4.7)	3.87 (−0.55–8.30, *P* = 0.085)	**4.46 (1.01**–**7.90, *P* = 0.012)**
Na (mmol/L)	139.5 (1.7)	0.69 (−0.55–1.93, *P* = 0.272)	−**1.12 (**−**2.19**–−**0.05, *P* = 0.040)**
HbA1C (mmol/mol)	37.5 (2.0)	1.86 (0.22–3.51, *P* = 0.027)	0.95 (−0.76–2.66, *P* = 0.271)
%FMD	2.5 (1.8)	−2.34 (−4.78–0.10, *P* = 0.060)	−3.15 (−6.33–0.04, *P* = 0.053)
6MWT Distance (metres)	925.4 (108.3)	108.41 (15.10–201.71, *P* = 0.024)	**145.60 (49.14**–**242.06, *P* = 0.004)**
Urea (log)	1.5 (0.2)	0.14 (−0.02–0.30, *P* = 0.094)	−0.03 (−0.18–0.12, *P* = 0.698)
Hb (g/L)	141.2 (13.5)	7.20 (−0.33–14.72, *P* = 0.060)	−3.73 (−8.61–1.16, *P* = 0.132)
Creatinine (μmol/L)	80.0 (16.3)	12.28 (4.29–20.26, *P* = 0.003)	0.15 (−4.01–4.30, *P* = 0.944)

This table shows the univariable, and multivariable regression analyses for both SARS-CoV-2 status (positive and negative) based on dependent variables at baseline after adjusting for relevant confounders (age, sex, BMI, baseline measure) for both the full dataset. (log), log transformed variable; 6MWT, 6-minute walk test; ABPM, ambulatory blood pressure; DBP, diastolic blood pressure; HbA1c, serum glycosylated haemoglobin; Na, serum sodium; SBP, systolic blood pressure; SD, standard deviation.

### Brachial flow mediated dilatation

Flow mediated dilatation (FMD) was statistically significantly lower at 12 months compared to baseline in the SARS-CoV-2 positive group (*P* = 0.039) but not in SARS-CoV-2 negative group (Fig. 2). Multivariable adjusted analysis for the FMD difference between baseline and 12 months showed decrease that missed statistical significance in the per-protocol dataset while the full dataset showed the results were concordant in direction and magnitude (full: –2.32% [–4.82 to 0.17], *P* = 0.067); per-protocol: –3.15% [–6.33 to 0.04], *P* = 0.053). (Table 3 and Table S6, Supplemental Digital Content).

### 6-Minute walk test

There was a significantly higher mean 6MWT distance (m) at 12 months compared to baseline in both groups (Fig. 2). Multivariable regression on the per-protocol group indicated that the SARS-CoV-2 positive group had a significantly greater increase in 6MWT distance by 146 m [49 to 242], *P* = 0.004) meters after adjusting for potential confounders compared to the SARS-CoV-2 negative group (Table 3).

### Laboratory outcomes

We carried out longitudinal analyses on laboratory variables included in the secondary outcomes. Haemoglobin, HbA1c and serum sodium showed nominally significant differences at baseline (Table 1) and in paired analysis but did not cross the multiple testing threshold for significance (Table 3).

### EQ-5D-3L

At baseline, participants who tested positive for SARS-CoV-2 reported significantly more problems across all EQ-5D-5L dimensions, except self-care, compared to those who tested negative. At 12 months, significant differences remained for mobility, usual activities, and pain, although these differences were attenuated, particularly in the per-protocol dataset (Tables S7 and S8, Supplemental Digital Content). EQ-5D-VAS and EQ-5D-Index scores were significantly lower in SARS-CoV-2 positive participants at baseline and remained lower at 12 months, despite slight declines in both groups over time (Tables S7 and S8, Supplemental Digital Content).

Linear regression models, adjusted for age and BMI, showed that SARS-CoV-2 positivity was associated with a significant reduction in EQ-5D-VAS at 12 months (–7.9, *P* < 0.001; Table S9, Supplemental Digital Content). However, after further adjustment for baseline values, SARS-CoV-2 positivity was no longer significantly associated with changes in EQ-5D-VAS. In contrast, a significant positive association with EQ-5D-Index was observed in both datasets (Table S9, Supplemental Digital Content). Logistic regression models assessing the likelihood of reporting problems in EQ-5D-5L dimensions at 12 months revealed that usual activities remained significantly affected in both datasets (Tables S10 and S11, Supplemental Digital Content). Figures S12 and S13, Supplemental Digital Content demonstrates the changes in EQ5D-VAS and EQ5D index over time from baseline to 12 months for both datasets.

## DISCUSSION

Our study demonstrates an increase in ABPM (SBP and DBP) over a 12-month period in participants who have recovered from COVID-19 infection compared to contemporaneous controls. This was associated with a reduction in FMD suggesting a potential vascular component for the observed BP changes. Additionally, there was an improvement in the 6MWT distance indicating improved physical function in the COVID-19 positive group relative to controls. Despite this improvement in physical function, quality of life parameters assessment revealed that even after 12 months recovery, the SARS-CoV-2 positive group had persisting impairment of perceived quality of life.

While there is growing evidence linking COVID-19 infection with new-onset hypertension, some studies have failed to show such an association. Therefore, a prospective study with careful adjudication of COVID-19 cases and state of the art vascular and blood pressure assessments has been needed to address this important question. Retrospective and observational studies often rely on self-reporting of symptoms, have small sample sizes, lack control groups, and involve short follow-up durations, necessitating cautious interpretation of findings [9]. For example, Akpek *et al.* observed elevated BPs in 18 out of 153 COVID-19 patients after a short follow up of 31.6 ± 5.0 days [2]. Another single centre prospective study reported no significant increase in BP after 3 and 6 months, although their sample size was small [10]. A cross-sectional observational study demonstrated over a quarter of COVID-19 positive adults reporting hypertension, but caution is warranted due to variability of cardiac related symptoms and study design [11]. In contrast, two longitudinal prospective studies showed that 29.7% and 33.3% of recovered COVID-19 patients developing hypertension at 1 year [12,13]. A cohort study involving 185 participants discharged 23 days following COVID-19 infection found that 40 (21.6%) had uncontrolled BP that required therapeutic change [3]. Gameil *et al.* observed elevated SBP in COVID-19 survivors (Control 120.63 ± 8.49 vs. Cases 126.70 ± 10.31) in univariate analysis (crude odds ratio 1.07 (1.03–1.109), *P* < 0.001) though this lost significance in the multivariate analysis [14]. In a recent retrospective observational study, Zhang *et al.* and colleagues observed higher incidence of new-onset hypertension in patients at 6 months in comparison to those with influenza [15].

A recent longitudinal cohort study of over 200 000 adults revealed a significant rise in new-onset hypertension during the COVID-19 pandemic, with the incidence rate increasing from 2.11 to 6.76 per 100 person-years, highlighting the need for widespread hypertension screening beyond individuals diagnosed with COVID-19 [16]. These findings underscore the need for further research with robust study designs and extended follow-up periods to elucidate the true relationship between COVID-19 and hypertension.

Our findings regarding FMD are consistent with a small study that found higher FMD in the control group compared to those with COVID-19 at a single visit [17]. Another study with 12 female participants (COVID-19 cases) age-matched with 11 controls without COVID-19 reported higher brachial BP (SBP 126 ± 19 vs.109 ± 8 mmHg; *P* = 0.010 and lower FMD (cases: 4.69 ± 2.68 vs. control: 5.73 ± 2.69%; *P* = 0.381) although the study was limited by its sample size [18]. These results align with our observation of reduced FMD in COVID-19 survivors, indicating persistent endothelial dysfunction. This may be associated, in part, to endothelial inflammation induced by SARS-CoV-2 [19]. In addition, the reduction in FMD could be also be an indirect marker of persistent lung injury following acute COVID-19 infection [20].

An exploratory observational study which was conducted by Kutz *et al.* in 43 matched SARS-CoV-2 participants with controls, they demonstrated that RAAS peptide concentrations (Angiotensin I, Angiotensin II, Angiotensin (1–5), Angiotensin (1–7), Plasma Renin Activity) were markedly lower in patients with COVID-19 with the enzymatic activity of ACE and ACE2 were not altered. In our study at baseline, RAAS fingerprinting results did not differ significantly between the two groups. Consequently, RAAS fingerprinting at 12 months was not repeated.

A recent meta-analysis provides evidence that severe COVID-19, but not mild COVID-19, is associated with increased blood glucose highlighting the importance for monitoring both BP and glycaemic control postinfection [21]. Another study indicated an increased risk of incident diabetes after hospital discharge post-COVID-19 infection, suggesting the need for ongoing blood glucose monitoring regardless of disease history and steroid treatment use [22]. Although our study did not demonstrate significant association with HbA1c, likely due to low statistical power, persistent hyperglycaemia in recovered COVID-19 individuals, even without a prior diabetes diagnosis, highlights the necessity for further longitudinal studies to determine the development of new-onset diabetes [23].

### Strengths and limitations

Our study presents several strengths compared to existing literature. We provide data at two separate time points (baseline and 12 months) and use of 24-h ABPM which is regarded as the reference method for diagnosing hypertension. In addition, 24-h ABPM is an objective measure and not susceptible to assessor or participant bias. Importantly, our study groups (SARS-CoV-2 negative and positive) did not have a prior history of hypertension or treatment with BP lowering drugs, limiting reducing potential confounding and allowing for tracking of longitudinal BP changes potentially attributable to COVID-19 infection. Additionally, %FMD was measured longitudinally as a marker of endothelial dysfunction providing valuable insights into vascular health in recovered COVID-19 individuals.

We acknowledge several limitations in our study. First, the LOCHIVNAR study was conducted at a single centre with a small sample size, which included an overrepresentation of females a factor that may have influenced the findings given established gender-based differences in vascular physiology [24,25]. While we accounted for sex as a covariate in multivariable models, future research should incorporate larger, more demographically balanced cohorts to ensure the generalisability of these results across diverse populations. The study treated all COVID-19 cases as a single group, but severity varied across participants. Hospitalisation status, ICU admission, ventilation, and antiviral treatments were not accounted for, which may have contributed to vascular differences. Future studies should incorporate stratification based on based on severity, hospital treatment, reinfections and vaccination status. Additionally, factors such as changes in lifestyle, medication use, or psychological stress during the pandemic may have confounded the relationship between COVID-19 and cardiovascular outcomes. While some findings (e.g., systolic BP *P* = 0.052, FMD *P* = 0.053) narrowly missed significance, the overall trends support a persistent cardiovascular impact of COVID-19. Given the study's exploratory nature, we recommend cautious interpretation and replication in larger datasets. Recruitment challenges, driven by the evolving dynamics of the pandemic – including vaccination rollouts and emerging variants – resulted in delays between COVID-19 diagnosis and baseline assessment. Participants were not screened for repeated infections and long COVID-19 symptoms were not quantified. Furthermore, data on the specific variants involved in the cases were not collected. Due to the pandemic, recruitment for research studies relied on an opt-in approach to minimize burden on participants who had COVID-19, potentially limiting sample diversity.

We also note that urinary albumin excretion was not measured in this study. The severity of cases was generally mild to moderate, which may influence the generalizability of the findings. To address this, a per-protocol analysis was conducted to ensure comparability between participants at baseline and at the 12-month follow-up. Recruitment bias may have occurred, as individuals with a strong interest in COVID-19 research were more likely to participate, potentially skewing the sample towards a more motivated subgroup. The study population may also lack sufficient diversity in terms of demographics and underlying health conditions, limiting the broader applicability of the conclusions. RAAS fingerprinting was not done at 12 months given the parameters at baseline were not significant.

Although quality of life (QoL) assessments were included, these subjective measures may not fully capture the complexity of post-COVID-19 recovery. Furthermore, despite rigorous quality control measures, measurement errors and variability in ambulatory blood pressure monitoring (ABPM) and flow-mediated dilation (FMD) could affect the internal validity of our results. RAAS fingerprinting was performed at baseline but not at 12 months due to funding constraints. Our data showed no significant RAAS differences between groups at baseline; however, longitudinal changes could provide mechanistic insights into COVID-19-related hypertension. While baseline RAAS parameters were similar between groups, future studies should explore longitudinal RAAS changes.

Though our study was restricted to patients who had a hospitalisation with COVID-19, our findings shed light on a potential longer-term complication of the infection on BP. Our results warrant further studies to understand the actual population impact which should be possible through analysis of real-world data. As the cardiovascular risk posed by BP is continuous, even a 2 mmHg change in SBP will translate to significant future population burden of cardiovascular disease [26]. Furthermore, if our results are validated, this will also necessitate changes in clinical practice requiring healthcare professionals to prioritise cardiovascular assessments to proactively address potential long-term complications. The observed improvements in 6MWT suggest a complex relationship between recovery and cardiometabolic health, adding value to our BP findings.

### Perspectives

As the COVID-19 pandemic continues to evolve, understanding its long-term health consequences is crucial. The LOCHINVAR study addresses a significant gap in the current literature by systematically investigating the prolonged cardiovascular effects of COVID-19. The LOCHINVAR study has implications for clinical practice, public health policy, and future research. Our study highlights the importance and will raise awareness about the potential long-term cardiovascular effects of COVID-19. It underscores the importance of long-term cardiovascular monitoring for COVID-19 survivors, vigilant screening and proactive management of hypertension, a focus on endothelial health and the need for validation and further research

## CONCLUSION

The LOCHINVAR study highlights a potential long-term cardiovascular risk in COVID-19 survivors, including elevated BP and endothelial dysfunction. While sample size and heterogeneity limit broad generalisability, our findings underscore the need for vigilant monitoring and further research. Future studies should integrate larger cohorts with stratified analyses based on disease severity and treatment exposure.

## ACKNOWLEDGEMENTS

We would like to thank all the participants from all sites who took part in this study. S.L. would like to acknowledge the efforts of Caitlin Gray and Susan Yip based at the Glasgow Clinical Research Facility who help supported the study.

Authorship criteria and contributions: All authors conceived and designed the project. SP, LM and SL wrote the protocol and obtained ethical approval. SL and SP analysed the data. SL wrote the first draft of the manuscript. All authors provided critical revisions to the final manuscript for submission.

### Conflicts of interest

For all authors there are no conflict of interest.

S.L., C.B. and S.P. are supported by Heart Research UK (RG2690/21/24). T.Q.B.T. is supported by a British Heart Foundation MBPhD Studentship (FS/MBPhD/22/28005). L.M. is supported by NHS Research Scotland Career Researcher Fellowship. S.P., C.B. and C.D. are supported by the British Heart Foundation Centre of Excellence Award (RE/18/6/34217) and the United Kingdom Research and Innovation Strength in Places Fund (SIPF00007/1). R.M.T. was supported by the BHF British Heart Foundation (RG/13/7/30099, RE/18/6/34217, CH/4/29762) and Canadian Institutes of Health Research (CRC 2021-00549/43881)

Funding received for this work: This study is funded by Heart Research UK (Registered Charity, No. 1044821, RG2690/21/24).

Potential conflicts of interest: None.

## Supplementary Material

Supplemental Digital Content
