# Supplementary material for: Long-term effects of SARS-CoV-2 infection on blood vessels and blood pressure – LOCHINVAR
Source: J Hypertens. 2025 Apr 10;43(6):1057–65. doi: 10.1097/HJH.0000000000004013 (PMC12052060; doi:10.1097/HJH.0000000000004013)

**Supplementary Methods**

**SARS-CoV-2 positive group**

The SARS-CoV-2 positive group included individuals that met the inclusion and exclusion criteria. The clinical criteria for clinically suspected COVID-19 include either fever, new onset cough and/or anosmia/dysgeusia and/or ≥2 of the following presenting features: fatigue/generalised weakness, headache, myalgia, sore throat/coryzal symptoms, breathlessness, anorexia/nausea/vomiting, diarrhoea, contact with known COVID-19 positive case. Patients who were SARS-CoV-2 RT-PCR positive and/or have diagnostic chest X-ray or computed tomogram (CT) features of COVID -19 which disclosed classic features of COVID-19 illness based on hospital radiological reporting was classified into the SARS-CoV-2 positive group. Eligible participants were invited to take part >12 weeks after discharge to allow for complete recovery from the underlying medical diagnosis that necessitated the hospital admission. However, as described below because of the changing nature of the pandemic, this ranged from 6 weeks to 28 weeks.

**SARS-CoV-2 negative group**

The SARS-CoV-2 negative group consisted of participants who were recruited from hospital admission records or through advertisement. Patients recruited from hospital admission records had been admitted with COVID-19-like symptoms but were SARS-CoV-2 RT-PCR negative and had chest X-ray or CT scans (if performed during the relevant admission) showing a low probability of COVID-19. Additionally, they were confirmed to be SARS-CoV-2 IgG antibody negative at enrolment. Participants recruited through advertisement had no history of SARS-CoV-2 infection or had previously tested negative by RT-PCR, and were confirmed to be SARS-CoV-2 IgG antibody negative at baseline enrolment.

**Clinical data collection**

Data were collected using CASTOR Electronic Data Capture (CASTOR EDC) (Castor, Amsterdam. www.castoredc.com) in an anonymised form using a unique study ID. ^9^ A link to the unique NHS Community Health Index (CHI) number was held separately on an NHS computer. Data on hospital admission variables was collected by a manual review of the patient case notes for the admission during the study period.

**Ethics and approvals**

This study was approved by the West of Scotland Research Ethics Committee 5 (21/WS/0075), Scotland, United Kingdom. The study is registered on Clinicaltrials.gov (NCT05087290) and UK Clinical Research Network; GN20CA501. Current protocol version 1.1 (24/06/2021). Written informed consent was given by each study participant. On review of the interim results at 12 months alongside the challenges in participant engagement with follow-up visits and the funding timelines the steering committee decided to cancel the planned 18-month follow-up visit and close the study after all the 12-month visits were completed. A substantial amendment was submitted and approved by West of Scotland Research Ethics Committee 5 (21/WS/0075), Scotland, United Kingdom on 31 May 2023.

**Supplementary Table(s)**

[Table S1 Overall Baseline Characteristics for Full and Per-Protocol Dataset (SARS-CoV-2 Negative Group vs SARS-CoV-2 Positive Group)](#_Toc186717482)

[Table S2 Baseline RAAS Fingerprinting for Per-Protocol and Full Data Set (SARS-CoV-2 Negative Group vs SARS-CoV-2 Positive Group)](#_Toc186717483)

[Table S3 Overall 12 month Characteristics for Full and Per-Protocol Dataset (SARS-CoV-2 Negative Group vs SARS-CoV-2 Positive Group)](#_Toc186717484)

[Table S4 Baseline Characteristics Univariable and Multivariable Regression Analyses](#_Toc186717485)

[Table S5 12 month Characteristics Univariable and Multivariable Regression Analyses](#_Toc186717486)

[Table S6 Longitudinal Regression Analyses - Full Dataset (Coefficient SARS-COV-2 Positive vs SARS-COV-2 Negative) at 12 months.](#_Toc186717487)

[Table S7 Overall Demographics – Full Dataset](#_Toc186717488)

[Table S8 Overall Demographics – Per Protocol Dataset](#_Toc186717489)

[Table S9 Adjusted Analyses for EQ5D VAS and EQ5D Index at 12 Months](#_Toc186717490)

[Table S10 Likelihood of reporting problems in EQ5DL Dimensions at baseline and at 12 months.](#_Toc186717491)

[Table S11 Changes in EQ5DL Dimensions from Baseline to 12 Months](#_Toc186717492)

Supplementary Figure(s)

[**Supplementary Figure 1 ABPM DBP Paired**](#_Toc186717493)

[Supplementary Figure 2 6-Minute Walk Test](#_Toc186717494)

[Supplementary Figure 3 Office SBP Paired](#_Toc186717495)

[Supplementary Figure 4 Office DBP Paired](#_Toc186717496)

[Supplementary Figure 5 Serum Sodium Paired](#_Toc186717497)

[Supplementary Figure 6 Serum HbA1c Paired](#_Toc186717498)

[Supplementary Figure 7 Serum Urea Paired](#_Toc186717499)

[Supplementary Figure 8 Serum Creatinine](#_Toc186717500)

[Supplementary Figure 9 Serum Haemoglobin (Hb) Paired](#_Toc186717501)

[Supplementary Figure 10 Urine Sodium Creatinine ratio (uNaCr) Paired](#_Toc186717502)

[Supplementary Figure 11 Urine Potassium Creatinine Ratio (uKCr) Paired](#_Toc186717503)

[Supplementary Figure 12 Changes in EQ5D-VAS Over Time](#_Toc186717504)

[Supplementary Figure 13 Changes in EQ5D-Index Over Time](#_Toc186717505)

Table S1 Overall Baseline Characteristics for Full and Per-Protocol Dataset (SARS-CoV-2 Negative Group vs SARS-CoV-2 Positive Group)

|  |  | **Full Dataset (n=97)** | | | **Per-Protocol Dataset (n=66)** | | |
| --- | --- | --- | --- | --- | --- | --- | --- |
| **Label**  **(Baseline)** | **levels** | **SARS-CoV-2 Neg**  **(n = 60)** | **SARS-CoV-2 Pos**  **(n = 37)** | **p** | **SARS-CoV-2 Neg**  **(n = 51)** | **SARS-CoV-2 Pos**  **(n = 15)** | **p** |
| Age  (years) | Median (IQR) | 49.5 (41.0 to 54.0) | 48.0 (44.0 to 54.0) | 0.680 | 50.0 (42.0 to 54.0) | 49.0 (43.0 to 53.5) | 0.939 |
| Sex  (n,%) | Female | 48 (80.0) | 19 (51.4) | 0.006 | 42 (82.4) | 6 (40.0) | **0.003** |
|  | Male | 12 (20.0) | 18 (48.6) |  | 9 (17.6) | 9 (60.0) |  |
| Ethnicity  (n,%) | Asian Indian | 1 (1.7) | 1 (2.7) | 0.196 |  |  |  |
|  | Asian Pakistani | 1 (1.7) | 2 (5.4) |  |  | 1 (6.7) |  |
|  | Caucasian | 58 (96.7) | 32 (86.5) |  |  |  |  |
|  | Afro-Carribean |  | 2 (5.4) |  |  |  |  |
| Smoking  (n,%) | Ever Smoker | 6 (10.0) | 7 (18.9) | 0.233 | 3 (5.9) | 1 (6.7) | 1.000 |
|  | Never Smoker | 54 (90.0) | 30 (81.1) |  | 48 (94.1) | 14 (93.3) |  |
| Alcohol  (n,%) | 0 | 28 (46.7) | 17 (45.9) | 0.712 | 24 (47.1) | 7 (46.7) | 1.000 |
|  | 1-14 | 30 (50.0) | 20 (54.1) |  | 25 (49.0) | 8 (53.3) |  |
|  | 14 | 2 (3.3) |  |  | 2 (3.9) |  |  |
| Office SBP  (mmHg) | Mean (SD) | 122.1 (12.8) | 123.6 (12.9) | 0.594 | 122.6 (13.2) | 122.8 (13.1) | 0.945 |
| Office DBP  (mmHg) | Mean (SD) | 75.4  (10.1) | 79.3 (9.6) | 0.066 | 74.8 (10.4) | 78.7 (5.6) | 0.169 |
| Year of Visit  (n, %) | 2020 | 7 (11.7) | 7 (18.9) | 0.112 |  |  |  |
|  | 2021 | 12 (20.0) | 2 (5.4) |  |  |  |  |
|  | 2022 | 41 (68.3) | 28 (75.7) |  | 39 (76.5) | 14 (93.3) |  |
| Days to Visit  (days) | Median (IQR) | 146.0 (120.8 to 167.0) | 190.0 (145.0 to 309.0) | <0.001 | 147.0 (127.0 to 167.0) | 147.0 (143.0 to 173.0) | 0.270 |
| Visit Interval  (days) | Median (IQR) | 371.5 (365.2 to 386.5) | 363.0 (356.0 to 369.0) | 0.002 | 370.0 (365.0 to 380.0) | 358.5 (356.0 to 366.0) | **0.002** |
| Vaccination Status  (n, %) | No | 2 (3.3) | 7 (18.9) | 0.013 | 1 (2.0) | 3 (20.0) | **0.034** |
|  | Yes | 58 (96.7) | 29 (78.4) |  | 50 (98.0) | 12 (80.0) |  |
|  | (Missing) | 0 (0.0) | 1 (2.7) |  | 4 (7.8) | 0 (0.0) |  |
|  | (Missing) | 0 (0.0) | 1 (2.7) |  | 4 (7.8) | 0 (0.0) |  |
| Height  (cm) | Mean (SD) | 1.7 (0.1) | 1.7 (0.1) | 0.115 | 1.7 (0.1) | 1.7 (0.1) | 0.075 |
| Weight  (kg) | Mean (SD) | 73.6 (16.5) | 80.8 (16.7) | 0.040 | 73.5 (16.4) | 82.1 (17.3) | 0.080 |
| BMI  (kg/m^2^) | Mean (SD) | 26.6 (5.0) | 28.2 (4.7) | 0.129 | 26.6 (4.9) | 27.9 (3.8) | 0.325 |
| Office SBP  (mmHg) | Mean (SD) | 122.1 (12.8) | 123.6 (12.9) | 0.594 | 122.6 (13.2) | 122.8 (13.1) | 0.945 |
| Office DBP  (mmHg) | Mean (SD) | 75.4 (10.1) | 79.3 (9.6) | 0.066 | 74.8 (10.4) | 78.7 (5.6) | 0.169 |
| Office Heart Rate  (bpm) | Mean (SD) | 66.9 (11.8) | 69.5 (11.4) | 0.290 | 65.4 (10.2) | 69.9 (11.3) | 0.155 |
| ABPM SBP  (mmHg) | Mean (SD) | 114.9 (9.8) | 119.4 (12.5) | 0.052 | 114.7 (10.0) | 117.8 (12.3) | 0.327 |
| ABPM DBP  (mmHg) | Mean (SD) | 73.1 (6.4) | 75.1 (7.3) | 0.148 | 73.3 (6.7) | 72.9 (4.6) | 0.827 |
| ABPM SBP (day)  (mmHg) | Mean (SD) | 117.9 (10.1) | 121.5 (13.0) | 0.127 | 117.6 (10.3) | 121.9 (12.0) | 0.177 |
| ABPM DBP(day)  (mmHg) | Mean (SD) | 75.6 (6.9) | 76.6 (8.4) | 0.514 | 75.5 (7.2) | 76.1 (4.5) | 0.760 |
| ABPM SBP (night) (mmHg) | Mean (SD) | 104.7 (10.1) | 108.6 (14.2) | 0.121 | 104.7 (10.1) | 105.3 (14.0) | 0.862 |
| ABPM DBP (night) (mmHg) | Mean (SD) | 64.6 (6.0) | 66.5 (9.4) | 0.223 | 64.9 (6.1) | 62.2 (5.8) | 0.137 |
| Hb  (g/L) | Mean (SD) | 135.2 (11.5) | 139.6 (13.0) | 0.082 | 134.6 (10.7) | 141.8 (12.9) | **0.033** |
| WBC  (x10^9/L) | Mean (SD) | 5.8 (1.4) | 5.8 (1.1) | 0.912 | 5.7 (1.3) | 5.7 (0.9) | 0.980 |
| Neutrophils  (x10^9/L) | Mean (SD) | 3.5 (1.2) | 3.4 (0.9) | 0.544 | 3.4 (1.2) | 3.4 (0.6) | 0.824 |
| Lymphocytes  (x10^9/L) | Mean (SD) | 1.7 (0.4) | 1.8 (0.6) | 0.241 | 1.7 (0.4) | 1.7 (0.5) | 0.642 |
| Na  (mmol/L) | Mean (SD) | 138.8 (1.8) | 140.2 (1.6) | <0.001 | 138.6 (1.8) | 140.4 (1.7) | **0.001** |
| K  (mmol/L) | Mean (SD) | 4.2 (0.3) | 4.2 (0.3) | 0.760 | 4.2 (0.3) | 4.2 (0.2) | 0.478 |
| Cl  (mmol/L) | Mean (SD) | 104.8 (2.0) | 104.6 (2.0) | 0.704 | 104.9 (2.0) | 104.8 (1.7) | 0.911 |
| Urea  (mmol/L) | Median (IQR) | 3.9 (3.4 to 4.6) | 4.7 (4.2 to 5.4) | 0.003 | 3.9 (3.5 to 4.7) | 4.9 (4.3 to 5.5) | **0.011** |
| Creatinine  (μmol/L) | Mean (SD) | 65.2 (13.0) | 73.3 (17.0) | 0.009 | 65.2 (11.7) | 75.7 (18.7) | **0.010** |
| Mg  (mmol/L) | Mean (SD) | 0.8 (0.1) | 0.8 (0.1) | 0.402 | 0.8 (0.1) | 0.9 (0.1) | 0.400 |
| Ca(adj)  (mmol/L) | Mean (SD) | 2.3 (0.1) | 2.3 (0.1) | 0.352 | 2.3 (0.1) | 2.3 (0.1) | 0.962 |
| Albumin  (g/L) | Mean (SD) | 40.9 (2.4) | 39.2 (6.9) | 0.089 | 40.9 (2.3) | 41.1 (1.8) | 0.819 |
| Bilirubin  (μmol/L) | Median (IQR) | 11.5 (9.0 to 14.2) | 10.0 (8.0 to 14.0) | 0.401 | 12.0 (9.0 to 14.5) | 10.0 (9.0 to 15.0) | 0.884 |
| ALT  (U/L) | Mean (SD) | 20.0 (11.7) | 23.6 (12.5) | 0.151 | 18.9 (10.7) | 21.7 (11.6) | 0.377 |
| Glucose  (mmol/L) | Mean (SD) | 8.7 (15.6) | 13.2 (20.8) | 0.241 | 9.3 (16.7) | 20.8 (26.8) | 0.052 |
| HbA1C  (mmol/mol) | Mean (SD) | 34.5 (2.6) | 37.1 (5.5) | 0.003 | 34.3 (2.6) | 37.1 (2.9) | **0.001** |
| Cholesterol  (mmol/L) | Mean (SD) | 3.6 (13.0) | 5.3 (1.0) | 0.441 | 3.4 (14.1) | 5.2 (0.6) | 0.616 |
| Triglyceride  (mmol/L) | Median (IQR) | 0.9 (0.7 to 1.1) | 1.1 (0.8 to 1.4) | 0.207 | 0.8 (0.7 to 1.1) | 0.8 (0.8 to 1.2) | 0.454 |
| HDL  (mmol/L) | Mean (SD) | 1.6 (0.5) | 1.5 (0.6) | 0.502 | 1.6 (0.5) | 1.7 (0.7) | 0.908 |
| uNaCr  (mmol/L) | Median (IQR) | 11.3 (8.7 to 15.6) | 11.9 (9.0 to 14.2) | 0.922 | 11.3 (8.7 to 15.6) | 9.4 (8.1 to 14.4) | 0.712 |
| uNaCr(log) | Mean (SD) | 2.5 (0.4) | 2.4 (0.3) | 0.816 | 2.5 (0.4) | 2.4 (0.4) | 0.736 |
| uKCr  (mmol/L) | Mean (SD) | 7.0 (2.2) | 5.6 (2.2) | 0.022 | 7.0 (2.2) | 5.6 (2.2) | 0.067 |
| uClCr (log) | Mean (SD) | 2.5 (0.4) | 2.4 (0.3) | 0.527 | 2.5 (0.4) | 2.4 (0.5) | 0.569 |
| %FMD | Median (IQR) | 4.7 (2.0 to 8.5) | 4.5 (2.4 to 6.5) | 0.461 | 4.7 (2.0 to 8.5) | 4.6 (2.6 to 6.8) | 0.662 |
| 6MWT Distance  (metres) | Median (IQR) | 654.0 (570.0 to 750.0) | 624.0 (544.5 to 727.5) | 0.656 | 654.0 (570.0 to 750.0) | 642.0 (534.0 to 747.0) | 0.800 |

This table presents the overall baseline demographics and clinical characteristics of the participants in the full dataset and per-protocol dataset stratified by SARS-CoV-2 status (positive or negative). Continuous variables are reported as mean (standard deviation), median (IQR) if data is skewed and does not follow a normal distribution and categorical variables are presented as frequency (percentage). P-values indicate group differences assessed using independent t-tests for continuous variables and chi-square tests for categorical variables.

SBP: Systolic Blood Pressure, DBP: Diastolic Blood Pressure, BMI: Body Mass Index, ABPM: Ambulatory Blood Pressure. Hb: Haemaglobin, WBC: White Blood Cell Count, Na: Serum Sodium, K: Serum Potassium, Cl: Serum Chloride, Ca(adj): Serum adjusted calcium, HbA1c: Serum glycosylated haemoglobin, HDL: Serum High-density lipoprotein (HDL) uNaCr: Urine Sodium Creatinine ratio, uKCr: Urine Potassium Creatinine Ratio; uClCr: Urine Chloride Creatinine Ratio, %FMD: percentage change in brachial flow mediated dilatation; 6MWT: 6 Minute Walk Test, (log): log transformed variable, SD: Standard Deviation, IQR: Interquartile range.

Table S2 Baseline RAAS Fingerprinting for Per-Protocol and Full Data Set (SARS-CoV-2 Negative Group vs SARS-CoV-2 Positive Group)

|  |  | **Full Dataset (n=97)** | | | **Per-Protocol Dataset (n=66)** | | |
| --- | --- | --- | --- | --- | --- | --- | --- |
| **Label**  **(Baseline)** | **levels** | **SARS-CoV-2 Neg**  **(n = 60)** | **SARS-CoV-2 Pos**  **(n = 37)** | **p** | **SARS-CoV-2 Neg**  **(n = 51)** | **SARS-CoV-2 Pos**  **(n = 15)** | **p** |
| Ang II (1-8) (pmol/L) | Median (IQR) | 80.1 (49.4 to 109.5) | 65.3 (39.5 to 111.6) | 0.491 | 71.7 (48.2 to 106.0) | 70.4 (45.2 to 111.1) | 0.941 |
| Ang 1-7 (pmol/L) | Median (IQR) | 2.5 (2.5 to 2.5) | 2.5 (2.5 to 2.5) | 0.927 |  |  |  |
| Ang I (1-10) (pmol/L) | Median (IQR) | 21.1 (14.1 to 32.2) | 16.3 (12.3 to 26.7) | 0.166 | 21.0 (14.3 to 30.6) | 19.8 (14.5 to 26.0) | 0.605 |
| Ang 1-5 (pmol/L) | Median (IQR) | 2.6 (1.5 to 3.6) | 3.0 (1.5 to 5.0) | 0.237 | 2.4 (1.5 to 3.4) | 2.9 (1.5 to 4.3) | 0.294 |
| Aldosterone (pmol/L) | Median (IQR) | 151.3 (109.1 to 223.2) | 181.8 (106.4 to 261.8) | 0.467 | 150.4 (105.5 to 223.3) | 147.4 (98.7 to 253.7) | 0.824 |
| AA2-Ratio (pmol/L) | Mean (SD) | 2.7 (2.2) | 3.3 (2.6) | 0.279 | 2.9 (2.3) | 3.1 (3.1) | 0.743 |
| PRA-S (pmol/L) | Median (IQR) | 110.1 (63.9 to 138.0) | 86.7 (49.9 to 138.3) | 0.375 | 94.6 (63.6 to 135.6) | 92.2 (59.8 to 137.1) | 0.915 |
| ACE-S (pmol/L) | Mean (SD) | 3.9 (2.1) | 4.5 (2.3) | 0.209 | 3.6 (1.3) | 4.1 (1.6) | 0.212 |
| AngII_1_8 (log) | Mean (SD) | 4.3 (0.6) | 4.2 (0.7) | 0.649 | 4.3 (0.6) | 4.3 (0.6) | 0.926 |
| Ang1_7 (log) | Mean (SD) | 0.8 (0.2) | 0.8 (0.2) | 0.848 |  |  |  |
| AngI_1_10 (log) | Mean (SD) | 3.0 (0.7) | 2.8 (0.8) | 0.154 | 3.0 (0.6) | 2.9 (0.5) | 0.477 |
| Ang1_5 (log) | Mean (SD) | 0.9 (0.6) | 1.1 (0.7) | 0.218 | 0.9 (0.5) | 1.0 (0.6) | 0.317 |
| PRA-S (log) | Mean (SD) | 4.6 (0.6) | 4.5 (0.7) | 0.490 | 4.5 (0.6) | 4.5 (0.6) | 0.845 |
| Aldosterone (log) | Mean (SD) | 5.1 (0.5) | 5.1 (0.7) | 0.552 | 5.1 (0.6) | 5.0 (0.6) | 0.585 |

This table presents the baseline RAAS Fingerprinting of the participants in the full dataset and per-protocol dataset stratified by SARS-CoV-2 status (positive or negative). Continuous variables are reported as mean (standard deviation), median (IQR) if data is skewed and does not follow a normal distribution and categorical variables are presented as frequency (percentage). P-values indicate group differences assessed using independent t-tests for continuous variables and chi-square tests for categorical variables.

AA2: Ratio of aldosterone/Ang II, ACE-S: Angiotensin converting enzyme, PRA-S: Angiotensin based markers for renin, (log): log transformed variable, SD: Standard Deviation, IQR: Interquartile range

Table S3 Overall 12 month Characteristics for Full and Per-Protocol Dataset (SARS-CoV-2 Negative Group vs SARS-CoV-2 Positive Group)

|  |  | **Full Dataset (n=97)** | | | **Per-protocol Dataset (n=66)** | | |
| --- | --- | --- | --- | --- | --- | --- | --- |
| **Label**  **(12 months)** | **Levels** | **SARS-CoV-2 Neg**  **n = 60** | **SARS-CoV-2 Pos**  **n = 37** | **p** | **SARS-CoV-2 Neg**  **n = 51** | **SARS-CoV-2 Pos**  **n = 15** | **p** |
| Age  (years) | Median (IQR) | 49.5 (41.0 to 54.0) | 48.0 (44.0 to 54.0) | 0.680 | 50.0 (42.0 to 54.0) | 49.0 (43.0 to 53.5) | 0.939 |
| Sex  (n, %) | Female | 48 (80.0) | 19 (51.4) | 0.006 | 42 (82.4) | 6 (40.0) | **0.003** |
|  | Male | 12 (20.0) | 18 (48.6) |  | 9 (17.6) | 9 (60.0) |  |
| Ethnicity  (n, %) | Asian Indian | 1 (1.7) | 1 (2.7) | 0.196 |  |  |  |
|  | Asian Pakistani | 1 (1.7) | 2 (5.4) |  |  | 1 (6.7) |  |
|  | Caucasian | 58 (96.7) | 32 (86.5) |  |  |  |  |
|  | Afro-Carribean |  | 2 (5.4) |  |  |  |  |
| Smoking  (n, %) | Ever Smoker | 6 (10.0) | 7 (18.9) | 0.233 | 3 (5.9) | 1 (6.7) | 1.000 |
|  | Never Smoker | 54 (90.0) | 30 (81.1) |  | 48 (94.1) | 14 (93.3) |  |
| Alcohol  (n, %) | 0 | 28 (46.7) | 17 (45.9) | 0.712 | 24 (47.1) | 7 (46.7) | 1.000 |
|  | 1-14 | 30 (50.0) | 20 (54.1) |  | 25 (49.0) | 8 (53.3) |  |
|  | 14 | 2 (3.3) |  |  | 2 (3.9) |  |  |
| Days to Visit (n, %) | Median (IQR) | 146.0 (120.8 to 167.0) | 190.0 (145.0 to 309.0) | <0.001 | 147.0 (127.0 to 167.0) | 147.0 (143.0 to 173.0) | 0.270 |
| Visit Interval (days) | Median (IQR) | 371.5 (365.2 to 386.5) | 363.0 (356.0 to 369.0) | 0.002 | 370.0 (365.0 to 380.0) | 358.5 (356.0 to 366.0) | **0.002** |
| Vaccination Status (n, %) | No | 2 (3.3) | 7 (18.9) | 0.013 | 1 (2.0) | 3 (20.0) | **0.034** |
|  | Yes | 58 (96.7) | 29 (78.4) |  | 50 (98.0) | 12 (80.0) |  |
|  | (Missing) | 0 (0.0) | 1 (2.7) |  |  |  |  |
| BMI (kg/m^2^) | Mean (SD) | 26.8 (4.9) | 27.7 (5.1) | 0.432 | 26.6 (4.5) | 27.9 (4.5) | 0.343 |
| Office SBP (mmHg) | Mean (SD) | 119.9 (12.8) | 127.8 (14.6) | 0.017 | 119.5 (13.2) | 128.0 (15.0) | 0.051 |
| Office DBP (mmHg) | Mean (SD) | 76.7 (8.9) | 80.5 (8.3) | 0.074 | 76.3 (8.9) | 81.2 (6.2) | 0.069 |
| ABPM SBP (mmHg) | Mean (SD) | 114.7 (9.5) | 121.6 (10.8) | 0.007 | 114.2 (9.5) | 123.9 (9.6) | **0.004** |
| ABPM DBP (mmHg) | Mean (SD) | 72.4 (6.8) | 75.3 (5.6) | 0.072 | 72.4 (6.9) | 76.3 (4.7) | 0.085 |
| ABPM SBP (day) (mmHg) | Mean (SD) | 117.2 (10.4) | 124.5 (11.2) | 0.008 | 116.9 (10.3) | 127.5 (10.4) | **0.004** |
| ABPM DBP (day) (mmHg) | Mean (SD) | 74.2 (7.9) | 77.6 (6.2) | 0.071 | 74.5 (7.3) | 78.7 (5.9) | 0.077 |
| ABPM SBP (night) (mmHg) | Mean (SD) | 104.0 (10.6) | 110.8 (10.7) | 0.012 | 104.0 (10.7) | 112.8 (9.3) | **0.015** |
| ABPM DBP(night) (mmHg) | Mean (SD) | 64.7 (7.6) | 66.5 (6.4) | 0.332 | 65.0 (7.7) | 67.5 (4.1) | 0.304 |
| Hb  (g/L) | Mean (SD) | 134.1 (12.0) | 140.4 (14.0) | 0.044 | 134.0 (11.6) | 141.2 (13.5) | 0.060 |
| WBC (x10^9/L) | Mean (SD) | 6.2 (1.4) | 5.6 (1.3) | 0.103 | 6.2 (1.5) | 5.5 (1.2) | 0.098 |
| Neutrophils (x10^9/L) | Mean (SD) | 3.8 (1.2) | 3.3 (1.0) | 0.125 | 3.8 (1.2) | 3.3 (0.9) | 0.150 |
| Lymphocytes (x10^9/L) | Mean (SD) | 1.8 (0.4) | 1.7 (0.4) | 0.205 | 1.8 (0.4) | 1.6 (0.4) | 0.203 |
| Na  (mmol/L) | Mean (SD) | 138.8 (2.1) | 139.6 (1.9) | 0.107 | 138.9 (2.1) | 139.5 (1.7) | 0.272 |
| K  (mmol/L) | Mean (SD) | 4.2 (0.3) | 4.2 (0.3) | 0.441 | 4.2 (0.3) | 4.2 (0.2) | 0.486 |
| Cl  (mmol/L) | Mean (SD) | 104.9 (2.2) | 105.2 (1.7) | 0.530 | 105.1 (2.2) | 105.2 (1.8) | 0.969 |
| Urea  (mmol/L) | Median (IQR) | 4.1 (3.3 to 4.8) | 5.2 (4.1 to 5.5) | 0.004 | 4.1 (3.3 to 4.8) | 5.2 (4.2 to 5.4) | **0.041** |
| Creatinine (μmol/L) | Mean (SD) | 67.2 (13.1) | 77.8 (15.3) | 0.002 | 67.7 (11.6) | 80.0 (16.3) | **0.003** |
| Mg  (mmol/L) | Mean (SD) | 0.9 (0.1) | 0.9 (0.1) | 0.302 | 0.9 (0.1) | 0.9 (0.0) | 0.983 |
| Ca(adj) (mmol/L) | Mean (SD) | 2.3 (0.1) | 2.4 (0.1) | 0.355 | 2.3 (0.1) | 2.4 (0.1) | 0.392 |
| Albumin (mmol/L) | Mean (SD) | 39.8 (5.5) | 41.2 (2.3) | 0.220 | 39.8 (5.8) | 41.8 (1.6) | 0.234 |
| Bilirubin (μmol/L) | Median (IQR) | 10.0 (8.0 to 12.0) | 11.0 (9.0 to 14.0) | 0.613 | 10.0 (8.0 to 12.5) | 11.0 (9.0 to 12.0) | 0.801 |
| ALT  (U/L) | Mean (SD) | 20.2 (10.5) | 25.7 (13.5) | 0.053 | 19.8 (10.6) | 26.5 (16.8) | 0.087 |
| Glucose (mmol/L) | Mean (SD) | 12.3 (22.1) | 19.0 (27.3) | 0.251 | 13.2 (23.4) | 20.5 (25.0) | 0.331 |
| HbA1C (mmol/mol) | Mean (SD) | 35.6 (2.8) | 37.5 (5.1) | 0.043 | 35.5 (2.9) | 37.5 (2.0) | **0.023** |
| Cholesterol (mmol/L) | Mean (SD) | 5.4 (1.0) | 5.3 (1.0) | 0.519 | 5.4 (1.0) | 5.4 (0.8) | 0.948 |
| Triglyceride (mmol/L) | Median (IQR) | 1.1 (0.8 to 1.5) | 1.2 (0.8 to 1.8) | 0.177 | 1.0 (0.8 to 1.3) | 1.1 (0.7 to 1.5) | 0.583 |
| HDL  (mmol/L) | Mean (SD) | 1.6 (0.4) | 1.4 (0.3) | 0.055 | 1.6 (0.4) | 1.4 (0.3) | 0.116 |
| Renin  (mIU/L) | Median (IQR) | 20.6 (11.8 to 30.9) | 19.1 (16.3 to 24.3) | 0.750 | 20.6 (11.7 to 29.2) | 21.6 (16.4 to 30.3) | 0.602 |
| Aldosterone (pmol/L) | Median (IQR) | 270.0 (191.2 to 365.2) | 298.5 (149.8 to 410.8) | 0.627 | 266.0 (189.8 to 355.8) | 255.0 (153.0 to 360.0) | 0.939 |
| NT-pro-BNP (ng/L) | Median (IQR) | 63.0 (42.0 to 96.0) | 49.0 (32.0 to 77.0) | 0.104 | 63.0 (41.0 to 91.0) | 38.0 (32.0 to 78.0) | 0.251 |
| uNaCr (mmol/L) | Median (IQR) | 11.8 (8.9 to 14.7) | 8.8 (7.5 to 10.7) | 0.007 | 11.9 (9.0 to 14.8) | 8.6 (7.5 to 9.2) | **0.014** |
| uKCr  (mmol/L) | Mean (SD) | 7.2 (3.4) | 5.9 (2.0) | 0.129 | 7.2 (3.2) | 5.1 (1.6) | 0.063 |
| uNaCr(log) | Mean (SD) | 2.3 (0.7) | 2.1 (0.4) | 0.167 | 2.3 (0.7) | 2.0 (0.5) | 0.253 |
| uClCr(log) | Mean (SD) | 2.5 (0.5) | 2.1 (0.4) | 0.032 | 2.4 (0.5) | 2.1 (0.4) | 0.081 |
| %FMD | Median (IQR) | 5.0 (2.6 to 7.4) | 2.4 (1.1 to 4.0) | 0.002 | 4.6 (2.6 to 7.0) | 1.8 (1.4 to 3.5) | 0.009 |
| 6MWT Distance (metres) | Median (IQR) | 750.0 (672.0 to 912.0) | 897.0 (816.0 to 1011.0) | 0.015 | 768.0 (708.0 to 930.0) | 900.0 (858.0 to 1002.0) | **0.022** |

This table presents the overall 12-month demographics and clinical characteristics of the participants in the full dataset and per-protocol dataset stratified by SARS-CoV-2 status (positive or negative). Continuous variables are reported as mean (standard deviation), median (IQR) if data is skewed and does not follow a normal distribution and categorical variables are presented as frequency (percentage). P-values indicate group differences assessed using independent t-tests for continuous variables and chi-square tests for categorical variables.

SBP: Systolic Blood Pressure, DBP: Diastolic Blood Pressure, BMI: Body Mass Index, ABPM: Ambulatory Blood Pressure. Hb: Haemaglobin, WBC: White Blood Cell Count, Na: Serum Sodium, K: Serum Potassium, Cl: Serum Chloride, Ca(adj): Serum adjusted calcium, HbA1c: Serum glycosylated haemoglobin, HDL: Serum High-density lipoprotein (HDL) uNaCr: Urine Sodium Creatinine ratio, uKCr: Urine Potassium Creatinine Ratio; uClCr: Urine Chloride Creatinine Ratio, %FMD: percentage change in brachial flow mediated dilatation; 6MWT: 6 Minute Walk Test, (log): log transformed variable, SD: Standard Deviation, IQR: Interquartile range.

Table S4 Baseline Characteristics Univariable and Multivariable Regression Analyses

|  | **Full Dataset** | | **Per-protocol Dataset** | |
| --- | --- | --- | --- | --- |
| **Dependent** | **Univariable** | **Multivariable** | **Univariable** | **Multivariable** |
| Office SBP (mmHg) | 1.43 (-3.88 to 6.75, p=0.594) | -0.46 (-5.81 to 4.88, p=0.864) | 0.27 (-7.48 to 8.02, p=0.945) | -1.33 (-9.45 to 6.79, p=0.744) |
| Office DBP (mmHg) | 3.87 (-0.26 to 7.99, p=0.066) | 2.25 (-1.87 to 6.37, p=0.281) | 3.90 (-1.70 to 9.51, p=0.169) | 2.40 (-3.30 to 8.10, p=0.403) |
| ABPM SBP (mmHg) | 4.48 (-0.03 to 9.00, p=0.052) | 1.41 (-2.88 to 5.70, p=0.515) | 3.05 (-3.13 to 9.24, p=0.327) | -1.15 (-6.96 to 4.66, p=0.694) |
| ABPM DBP (mmHg) | 2.07 (-0.74 to 4.88, p=0.148) | 0.87 (-2.00 to 3.74, p=0.549) | -0.41 (-4.12 to 3.31, p=0.827) | -2.54 (-6.33 to 1.25, p=0.185) |
| ABPM SBP (day) (mmHg) | 3.62 (-1.05 to 8.30, p=0.127) | 1.06 (-3.47 to 5.59, p=0.644) | 4.28 (-1.98 to 10.54, p=0.177) | 0.56 (-5.57 to 6.70, p=0.855) |
| ABPM DBP (day) (mmHg) | 1.03 (-2.08 to 4.14, p=0.514) | 0.26 (-2.97 to 3.50, p=0.872) | 0.60 (-3.32 to 4.53, p=0.760) | -1.06 (-5.23 to 3.11, p=0.613) |
| ABPM SBP (night) (mmHg) | 3.93 (-1.06 to 8.93, p=0.121) | 0.72 (-3.96 to 5.40, p=0.761) | 0.57 (-5.99 to 7.14, p=0.862) | -4.09 (-10.03 to 1.85, p=0.174) |
| ABPM DBP (night) (mmHg) | 1.94 (-1.20 to 5.08, p=0.223) | 1.02 (-2.21 to 4.25, p=0.532) | -2.70 (-6.28 to 0.88, p=0.137) | -4.30 (-8.02 to -0.58, p=0.024) |
| Hb  (g/L) | 4.45 (-0.57 to 9.47, p=0.082) | -1.26 (-5.19 to 2.67, p=0.526) | 7.21 (0.62 to 13.80, p=0.033) | -0.95 (-6.10 to 4.20, p=0.713) |
| WBC  (x10^9/L) | 0.03 (-0.50 to 0.56, p=0.912) | -0.01 (-0.56 to 0.54, p=0.968) | 0.01 (-0.68 to 0.70, p=0.980) | 0.17 (-0.59 to 0.93, p=0.657) |
| Neutrophils (x10^9/L) | -0.14 (-0.61 to 0.32, p=0.544) | -0.13 (-0.61 to 0.36, p=0.602) | -0.07 (-0.70 to 0.56, p=0.824) | 0.12 (-0.57 to 0.81, p=0.731) |
| Lymphocytes (x10^9/L) | 0.12 (-0.08 to 0.32, p=0.241) | 0.09 (-0.11 to 0.30, p=0.372) | 0.06 (-0.18 to 0.29, p=0.642) | 0.07 (-0.20 to 0.34, p=0.610) |
| Na  (mmol/L) | 1.40 (0.66 to 2.13, p<0.001) | 1.22 (0.44 to 2.00, p=0.002) | 1.81 (0.76 to 2.86, p=0.001) | 1.57 (0.41 to 2.74, p=0.009) |
| K  (mmol/L) | -0.02 (-0.13 to 0.09, p=0.760) | -0.03 (-0.15 to 0.08, p=0.550) | -0.05 (-0.20 to 0.09, p=0.478) | -0.05 (-0.21 to 0.11, p=0.518) |
| Cl  (mmol/L) | -0.16 (-1.00 to 0.68, p=0.704) | -0.03 (-0.89 to 0.83, p=0.947) | -0.06 (-1.19 to 1.06, p=0.911) | 0.39 (-0.78 to 1.55, p=0.509) |
| Urea (log) | 0.17 (0.06 to 0.29, p=0.003) | 0.12 (0.01 to 0.24, p=0.037) | 0.20 (0.05 to 0.34, p=0.009) | 0.09 (-0.06 to 0.24, p=0.222) |
| Creatinine  (μmol/L) | 8.11 (2.03 to 14.19, p=0.009) | 1.48 (-3.41 to 6.36, p=0.550) | 10.54 (2.61 to 18.46, p=0.010) | 0.20 (-5.77 to 6.16, p=0.948) |
| Ca(adj) (mmol/L) | 0.02 (-0.02 to 0.05, p=0.352) | 0.01 (-0.02 to 0.05, p=0.506) | 0.00 (-0.05 to 0.05, p=0.962) | 0.01 (-0.04 to 0.06, p=0.768) |
| Glucose  (mmol/L) | 4.45 (-3.05 to 11.95, p=0.241) | 0.99 (-6.81 to 8.79, p=0.802) | 11.49 (-0.10 to 23.07, p=0.052) | 7.04 (-5.43 to 19.51, p=0.263) |
| HbA1C  (mmol/mol) | 2.53 (0.87 to 4.20, p=0.003) | 1.97 (0.27 to 3.67, p=0.024) | 2.79 (1.22 to 4.35, p=0.001) | 2.24 (0.68 to 3.80, p=0.006) |
| Cholesterol (mmol/L) | 1.71 (-2.67 to 6.08, p=0.441) | 0.67 (-3.94 to 5.27, p=0.775) | 1.84 (-5.46 to 9.15, p=0.616) | 0.61 (-7.28 to 8.50, p=0.878) |
| Triglyceride (mmol/L) | 0.15 (-0.07 to 0.36, p=0.170) | 0.04 (-0.18 to 0.26, p=0.731) | 0.08 (-0.16 to 0.31, p=0.526) | -0.04 (-0.27 to 0.20, p=0.749) |
| HDL  (mmol/L) | -0.08 (-0.30 to 0.15, p=0.502) | -0.01 (-0.24 to 0.22, p=0.937) | 0.02 (-0.31 to 0.35, p=0.908) | 0.12 (-0.24 to 0.47, p=0.524) |
| Renin(log) | 0.11 (-0.15 to 0.37, p=0.398) | 0.14 (-0.13 to 0.41, p=0.306) | 0.17 (-0.16 to 0.50, p=0.312) | 0.15 (-0.20 to 0.50, p=0.407) |
| Aldosterone(cat) | 0.04 (-0.17 to 0.25, p=0.721) | -0.01 (-0.23 to 0.22, p=0.961) | 0.03 (-0.27 to 0.33, p=0.824) | 0.06 (-0.26 to 0.39, p=0.710) |
| NT-pro-BNP (log) | -0.01 (-0.31 to 0.29, p=0.935) | 0.09 (-0.22 to 0.39, p=0.563) | -0.17 (-0.55 to 0.21, p=0.367) | -0.07 (-0.48 to 0.34, p=0.739) |
| Ang II (1-8) (log) | -0.06 (-0.35 to 0.22, p=0.649) | 0.03 (-0.25 to 0.31, p=0.844) | -0.02 (-0.39 to 0.36, p=0.926) | -0.02 (-0.40 to 0.37, p=0.929) |
| Ang 1-7 (log) | -0.01 (-0.10 to 0.08, p=0.848) | -0.01 (-0.11 to 0.09, p=0.866) | -0.00 (-0.06 to 0.06, p=0.978) | -0.00 (-0.07 to 0.06, p=0.940) |
| Ang I (1-10) (log) | -0.23 (-0.54 to 0.09, p=0.154) | -0.14 (-0.46 to 0.18, p=0.395) | -0.13 (-0.47 to 0.22, p=0.477) | -0.08 (-0.44 to 0.28, p=0.647) |
| Ang 1-5 (log) | 0.17 (-0.10 to 0.43, p=0.218) | 0.21 (-0.07 to 0.48, p=0.134) | 0.15 (-0.15 to 0.46, p=0.317) | 0.05 (-0.25 to 0.36, p=0.730) |
| PRA-S (log) | -0.10 (-0.37 to 0.18, p=0.490) | -0.00 (-0.28 to 0.27, p=0.972) | -0.04 (-0.40 to 0.32, p=0.845) | -0.03 (-0.39 to 0.34, p=0.889) |
| Aldosterone (log) | 0.08 (-0.19 to 0.35, p=0.552) | 0.11 (-0.17 to 0.40, p=0.432) | -0.09 (-0.43 to 0.25, p=0.585) | -0.09 (-0.47 to 0.29, p=0.634) |
| AA2-Ratio (pmol/L) | 0.55 (-0.45 to 1.55, p=0.279) | 0.24 (-0.79 to 1.26, p=0.649) | 0.25 (-1.26 to 1.76, p=0.743) | 0.01 (-1.61 to 1.62, p=0.992) |
| ACE-S (pmol/L) | 0.60 (-0.34 to 1.53, p=0.209) | 0.57 (-0.43 to 1.57, p=0.258) | 0.50 (-0.30 to 1.30, p=0.212) | 0.35 (-0.53 to 1.23, p=0.429) |
| uNaCr (log) | -0.02 (-0.21 to 0.17, p=0.816) | 0.05 (-0.16 to 0.26, p=0.646) | -0.04 (-0.31 to 0.22, p=0.736) | 0.08 (-0.19 to 0.35, p=0.555) |
| uKCr (mmol/L) | -1.35 (-2.49 to -0.20, p=0.022) | -0.17 (-1.31 to 0.96, p=0.761) | -1.41 (-2.93 to 0.10, p=0.067) | -0.53 (-2.02 to 0.96, p=0.477) |
| uClCr (log) | -0.06 (-0.24 to 0.12, p=0.527) | 0.02 (-0.18 to 0.23, p=0.806) | -0.07 (-0.33 to 0.19, p=0.569) | 0.06 (-0.21 to 0.33, p=0.648) |
| %FMD | -6.76 (-14.75 to 1.24, p=0.096) | -5.78 (-14.42 to 2.86, p=0.186) | -6.30 (-17.37 to 4.76, p=0.259) | -4.18 (-16.39 to 8.04, p=0.496) |
| 6MWT Distance (metres) | -10.74 (-77.22 to 55.73, p=0.748) | -16.19 (-88.57 to 56.19, p=0.657) | -10.59 (-98.04 to 76.86, p=0.810) | -27.91 (-121.88 to 66.06, p=0.555) |

This table shows the univariable, and multivariable regression analyses based on dependent variables at baseline for both the full and per-protocol dataset.

SBP: Systolic Blood Pressure, DBP: Diastolic Blood Pressure, BMI: Body Mass Index, ABPM: Ambulatory Blood Pressure. Hb: Haemaglobin, WBC: White Blood Cell Count, Na: Serum Sodium, K: Serum Potassium, Cl: Serum Chloride, Ca(adj): Serum adjusted calcium, HbA1c: Serum glycosylated haemoglobin, HDL: Serum High-density lipoprotein (HDL), AA2: Ratio of aldosterone/Ang II, ACE-S: Angiotensin converting enzyme, PRA-S: Angiotensin based markers for renin, uNaCr: Urine Sodium Creatinine ratio, uKCr: Urine Potassium Creatinine Ratio; uClCr: Urine Chloride Creatinine Ratio, %FMD: percentage change in brachial flow mediated dilatation; 6MWT: 6 Minute Walk Test, (log): log transformed variable, SD: Standard Deviation, IQR: Interquartile range.

Table S5 12 month Characteristics Univariable and Multivariable Regression Analyses

|  | **Full Dataset** | | **Per-protocol Dataset** | |
| --- | --- | --- | --- | --- |
| **Dependent (12 months)** | **Univariable** | **Multivariable** | **Univariable** | **Multivariable** |
| Office SBP (mmHg) | 7.91 (1.43 to 14.39, p=0.017) | 5.73 (-1.15 to 12.62, p=0.101) | 8.50 (-0.02 to 17.02, p=0.051) | 4.94 (-4.09 to 13.96, p=0.278) |
| Office DBP (mmHg) | 3.81 (-0.38 to 8.00, p=0.074) | 2.33 (-2.26 to 6.92, p=0.315) | 4.88 (-0.40 to 10.16, p=0.069) | 2.32 (-3.38 to 8.02, p=0.419) |
| ABPM SBP (mmHg) | 6.94 (1.99 to 11.90, p=0.007) | 3.86 (-1.47 to 9.19, p=0.153) | 9.71 (3.30 to 16.12, p=0.004) | 5.38 (-1.96 to 12.71, p=0.147) |
| ABPM DBP (mmHg) | 2.98 (-0.27 to 6.22, p=0.072) | 2.22 (-1.51 to 5.96, p=0.239) | 3.87 (-0.55 to 8.30, p=0.085) | 2.81 (-2.59 to 8.21, p=0.301) |
| ABPM SBP (day) (mmHg) | 7.35 (2.00 to 12.69, p=0.008) | 4.18 (-1.68 to 10.04, p=0.159) | 10.54 (3.61 to 17.48, p=0.004) | 5.39 (-2.61 to 13.39, p=0.182) |
| ABPM DBP (day) (mmHg) | 3.41 (-0.30 to 7.13, p=0.071) | 2.79 (-1.53 to 7.10, p=0.202) | 4.26 (-0.48 to 9.00, p=0.077) | 2.80 (-2.94 to 8.54, p=0.332) |
| ABPM SBP (night) (mmHg) | 6.82 (1.52 to 12.13, p=0.012) | 3.47 (-2.40 to 9.33, p=0.242) | 8.86 (1.82 to 15.91, p=0.015) | 4.40 (-3.73 to 12.52, p=0.283) |
| ABPM DBP (night) (mmHg) | 1.77 (-1.85 to 5.39, p=0.332) | 0.36 (-3.81 to 4.54, p=0.863) | 2.50 (-2.33 to 7.33, p=0.304) | 1.09 (-4.79 to 6.97, p=0.712) |
| Hb  (g/L) | 6.28 (0.17 to 12.40, p=0.044) | -3.98 (-8.78 to 0.81, p=0.102) | 7.20 (-0.33 to 14.72, p=0.060) | -5.65 (-11.49 to 0.19, p=0.058) |
| WBC  (x10^9/L) | -0.56 (-1.24 to 0.12, p=0.103) | -0.43 (-1.18 to 0.32, p=0.254) | -0.75 (-1.64 to 0.14, p=0.098) | -0.54 (-1.57 to 0.50, p=0.304) |
| Neutrophils (x10^9/L) | -0.43 (-0.97 to 0.12, p=0.125) | -0.30 (-0.90 to 0.30, p=0.329) | -0.53 (-1.25 to 0.20, p=0.150) | -0.36 (-1.18 to 0.46, p=0.389) |
| Lymphocytes (x10^9/L) | -0.13 (-0.34 to 0.07, p=0.205) | -0.12 (-0.35 to 0.11, p=0.310) | -0.17 (-0.44 to 0.10, p=0.203) | -0.14 (-0.46 to 0.18, p=0.387) |
| Na  (mmol/L) | 0.81 (-0.18 to 1.79, p=0.107) | 0.36 (-0.74 to 1.46, p=0.521) | 0.69 (-0.55 to 1.93, p=0.272) | 0.17 (-1.26 to 1.60, p=0.813) |
| K  (mmol/L) | 0.05 (-0.08 to 0.19, p=0.441) | -0.00 (-0.16 to 0.15, p=0.972) | 0.06 (-0.11 to 0.22, p=0.486) | -0.02 (-0.21 to 0.16, p=0.814) |
| Cl  (mmol/L) | 0.32 (-0.68 to 1.31, p=0.530) | 0.20 (-0.93 to 1.34, p=0.723) | 0.03 (-1.31 to 1.37, p=0.969) | -0.03 (-1.60 to 1.53, p=0.965) |
| Urea (log) | 0.19 (0.06 to 0.32, p=0.004) | 0.09 (-0.05 to 0.22, p=0.215) | 0.14 (-0.02 to 0.30, p=0.094) | -0.01 (-0.18 to 0.16, p=0.913) |
| Creatinine  (μmol/L) | 8.11 (2.03 to 14.19, p=0.009) | 1.48 (-3.41 to 6.36, p=0.550) | 10.54 (2.61 to 18.46, p=0.010) | 0.20 (-5.77 to 6.16, p=0.948) |
| Ca(adj)  (mmol/L) | 0.02 (-0.02 to 0.05, p=0.352) | 0.01 (-0.02 to 0.05, p=0.506) | 0.00 (-0.05 to 0.05, p=0.962) | 0.01 (-0.04 to 0.06, p=0.768) |
| Glucose  (mmol/L) | 4.45 (-3.05 to 11.95, p=0.241) | 0.99 (-6.81 to 8.79, p=0.802) | 11.49 (-0.10 to 23.07, p=0.052) | 7.04 (-5.43 to 19.51, p=0.263) |
| HbA1C  (mmol/mol) | 2.53 (0.87 to 4.20, p=0.003) | 1.97 (0.27 to 3.67, p=0.024) | 2.79 (1.22 to 4.35, p=0.001) | 2.24 (0.68 to 3.80, p=0.006) |
| Cholesterol (mmol/L) | 1.71 (-2.67 to 6.08, p=0.441) | 0.67 (-3.94 to 5.27, p=0.775) | 1.84 (-5.46 to 9.15, p=0.616) | 0.61 (-7.28 to 8.50, p=0.878) |
| Triglyceride (log) | 0.18 (-0.05 to 0.42, p=0.124) | 0.13 (-0.12 to 0.39, p=0.290) | 0.13 (-0.17 to 0.42, p=0.398) | 0.10 (-0.21 to 0.42, p=0.515) |
| HDL  (mmol/L) | -0.08 (-0.30 to 0.15, p=0.502) | -0.01 (-0.24 to 0.22, p=0.937) | 0.02 (-0.31 to 0.35, p=0.908) | 0.12 (-0.24 to 0.47, p=0.524) |
| Renin (log) | 0.11 (-0.15 to 0.37, p=0.398) | 0.14 (-0.13 to 0.41, p=0.306) | 0.17 (-0.16 to 0.50, p=0.312) | 0.15 (-0.20 to 0.50, p=0.407) |
| Aldosterone (cat) | 0.04 (-0.17 to 0.25, p=0.721) | -0.01 (-0.23 to 0.22, p=0.961) | 0.03 (-0.27 to 0.33, p=0.824) | 0.06 (-0.26 to 0.39, p=0.710) |
| NT-pro-BNP (log) | -0.01 (-0.31 to 0.29, p=0.935) | 0.09 (-0.22 to 0.39, p=0.563) | -0.17 (-0.55 to 0.21, p=0.367) | -0.07 (-0.48 to 0.34, p=0.739) |
| uNaCr (log) | -0.24 (-0.58 to 0.10, p=0.167) | -0.17 (-0.55 to 0.21, p=0.381) | -0.29 (-0.79 to 0.21, p=0.253) | -0.05 (-0.64 to 0.55, p=0.873) |
| uKCr  (mmol/L) | -1.30 (-3.00 to 0.39, p=0.129) | -0.88 (-2.60 to 0.84, p=0.309) | -2.09 (-4.29 to 0.11, p=0.063) | -1.11 (-3.57 to 1.34, p=0.365) |
| uClCr  (log) | -0.31 (-0.59 to -0.03, p=0.032) | -0.25 (-0.56 to 0.06, p=0.106) | -0.34 (-0.73 to 0.04, p=0.081) | -0.13 (-0.57 to 0.32, p=0.572) |
| %FMD | -2.51 (-4.07 to -0.95, p=0.002) | -1.98 (-3.92 to -0.04, p=0.046) | -2.52 (-4.45 to -0.59, p=0.012) | -2.90 (-5.49 to -0.31, p=0.029) |
| 6MWT Distance (metres) | 109.25 (16.98 to 201.52, p=0.021) | 126.39 (18.64 to 234.14, p=0.022) | 118.45 (21.01 to 215.90, p=0.018) | 153.86 (41.29 to 266.44, p=0.008) |

This table shows the univariable, and multivariable regression analyses based on dependent variables at 12-months for both the full and per-protocol dataset.

SBP: Systolic Blood Pressure, DBP: Diastolic Blood Pressure, BMI: Body Mass Index, ABPM: Ambulatory Blood Pressure. Hb: Haemaglobin, WBC: White Blood Cell Count, Na: Serum Sodium, K: Serum Potassium, Cl: Serum Chloride, Ca(adj): Serum adjusted calcium, HbA1c: Serum glycosylated haemoglobin, HDL: Serum High-density lipoprotein (HDL) uNaCr: Urine Sodium Creatinine ratio, uKCr: Urine Potassium Creatinine Ratio; uClCr: Urine Chloride Creatinine Ratio, %FMD: percentage change in brachial flow mediated dilatation; 6MWT: 6 Minute Walk Test, (log): log transformed variable, SD: Standard Deviation, IQR: Interquartile range.

Table S6 Longitudinal Regression Analyses - Full Dataset (Coefficient SARS-COV-2 Positive vs SARS-COV-2 Negative) at 12 months.

| **Full Dataset** | **Mean (SD)** | **Univariable model** | **Multivariable (age, sex, BMI, baseline measure)** |
| --- | --- | --- | --- |
| Office SBP  (mmHg) | 127.8 (14.6) | 7.91 (1.43 to 14.39, p=0.017) | 6.30 (0.81 to 11.79, p=0.025) |
| Office DBP  (mmHg) | 80.5 (8.3) | 3.81 (-0.38 to 8.00, p=0.074) | 1.86 (-1.46 to 5.19, p=0.267) |
| ABPM SBP  (mmHg) | 121.6 (10.8) | 6.94 (1.99 to 11.90, p=0.007) | 2.63 (-1.11 to 6.37, p=0.165) |
| ABPM DBP  (mmHg) | 75.3 (5.6) | 2.98 (-0.27 to 6.22, p=0.072) | 2.30 (-0.39 to 4.99, p=0.092) |
| Na (mmol/L) | 139.6 (1.9) | 0.81 (-0.18 to 1.79, p=0.107) | -0.67 (-1.55 to 0.20, p=0.128) |
| HbA1c (mmol/mol) | 37.5 (5.1) | 1.73 (-0.04 to 3.50, p=0.056) | 0.40 (-0.86 to 1.65, p=0.529) |
| %FMD | 2.5 (2.1) | -2.35 (-4.27 to -0.43, p=0.018) | -2.32 (-4.82 to 0.17, p=0.067) |
| 6MWT Distance (metres) | 919.6 (138.1) | 102.66 (23.82 to 181.50, p=0.012) | 132.13 (48.79 to 215.48, p=0.002) |
| Urea (log) | 1.6 (0.3) | 0.19 (0.06 to 0.32, p=0.004) | 0.05 (-0.07 to 0.16, p=0.417) |
| Hb (g/L) | 140.4 (14.0) | 6.28 (0.17 to 12.40, p=0.044) | -1.06 (-4.67 to 2.54, p=0.559) |
| uNaCr  (mmol/L) | 9.2 (3.0) | -2.04 (-4.97 to 0.88, p=0.166) | -1.31 (-4.33 to 1.71, p=0.385) |
| uKCr  (mmol/L) | 6.2 (2.1) | -0.82 (-2.75 to 1.11, p=0.397) | -0.08 (-1.93 to 1.76, p=0.926) |
| Creatinine  (μmol/L) | 77.8 (15.3) | 10.61 (3.91 to 17.30, p=0.002) | 0.15 (-2.95 to 3.24, p=0.926) |

This table shows the univariable, and multivariable regression analyses for both SARS-CoV-2 status (positive and negative) based on dependent variables at baseline after adjusting for relevant confounders (age, sex, BMI, baseline measure) for both the full dataset.

SBP: Systolic Blood Pressure, DBP: Diastolic Blood Pressure, BMI: Body Mass Index, ABPM: Ambulatory Blood Pressure. Hb: Haemaglobin, WBC: White Blood Cell Count, Na: Serum Sodium,:HbA1c: Serum glycosylated haemoglobin, %FMD: % change in brachial flow mediated dilatation, 6MWT: 6 minute walk test, uNaCr: Urine Sodium Creatinine ratio, uKCr: Urine Potassium Creatinine Ratio, ((log): log transformed variable, SD: Standard Deviation.

Table S7 Overall Demographics – Full Dataset

This table presents the baseline demographic and clinical characteristics of participants in the full dataset, stratified by SARS-CoV-2 status (positive or negative). Continuous variables are reported as mean (standard deviation), and categorical variables are presented as frequency (percentage). P-values indicate group differences assessed using independent t-tests for continuous variables and chi-square tests for categorical variables.

|  | | **Baseline** | | | **12 months** | | |  |  |
| --- | --- | --- | --- | --- | --- | --- | --- | --- | --- |
| label | levels | SARS-CoV-2  Negative | SARS-CoV-2  Positive | P | SARS-CoV-2  Negative | SARS-CoV-2  Positive | P | |  |
| Age (years) | Mean SD) | 47.9 (7.5) | 48.6 (6.7) | 0.221 |  |  |  | |  |
| Sex (n, %) | Female | 288 (80.0) | 114 (51.4) | <0.001 |  |  |  | |  |
|  | Male | 72 (20.0) | 108 (48.6) |  |  |  |  | |  |
| BMI (kg/m^2^) | Mean SD) | 26.6 (5.0) | 28.2 (4.7) | <0.001 |  |  |  | |  |
| Mobility | No problems | 339 (94.2) | 177 (79.7) | <0.001 | 328 (91.1) | 179 (80.6) | 0.008 | |  |
|  | Some Problems | 20 (5.6) | 44 (19.8) |  | 25 (6.9) | 30 (13.5) |  | |  |
|  | (Missing) | 1 (0.3) | 1 (0.5) |  | 7 (1.9) | 13 (5.9) |  | |  |
| Self-care | No problems | 359 (99.7) | 215 (96.8) | - | 318 (88.3) | 144 (64.9) | - | |  |
|  | Some Problems | 0 (0.0) | 6 (2.7) |  | 0 (0.0) | 0 (0.0) |  | |  |
|  | (Missing) | 1 (0.3) | 1 (0.5) |  | 42 (11.7) | 78 (35.1) |  | |  |
| Activity | No problems | 340 (94.4) | 160 (72.1) | <0.001 | 314 (87.2) | 167 (75.2) | 0.004 | |  |
|  | Some Problems | 19 (5.3) | 55 (24.8) |  | 39 (10.8) | 42 (18.9) |  | |  |
|  | A lot of Problems | 0 (0.0) | 6 (2.7) |  | 0 (0.0) | 0 (0.0) |  | |  |
|  | (Missing) | 1 (0.3) | 1 (0.5) |  | 7 (1.9) | 13 (5.9) |  | |  |
| Pain | No problems | 352 (97.8) | 147 (66.2) | <0.001 | 279 (77.5) | 148 (66.7) | 0.005 | |  |
|  | Some Problems | 0 (0.0) | 68 (30.6) |  | 68 (18.9) | 61 (27.5) |  | |  |
|  | A lot of Problems | 7 (1.9) | 6 (2.7) |  | 6 (1.7) | 0 (0.0) |  | |  |
|  | (Missing) | 1 (0.3) | 1 (0.5) |  | 7 (1.9) | 13 (5.9) |  | |  |
| Anxiety | No problems | 347 (96.4) | 148 (66.7) | <0.001 | 287 (79.7) | 162 (73.0) | 0.052 | |  |
|  | Some Problems | 12 (3.3) | 67 (30.2) |  | 60 (16.7) | 47 (21.2) |  | |  |
|  | A lot of Problems | 0 (0.0) | 6 (2.7) |  | 6 (1.7) | 0 (0.0) |  | |  |
|  | (Missing) | 1 (0.3) | 1 (0.5) |  | 7 (1.9) | 13 (5.9) |  | |  |
| EQ5D_VAS | | Mean (SD) | 85.4 (11.3) | 74.0 (15.3) | <0.001 | 82.0 (15.3) | 74.9 (15.6) | <0.001 | |
| EQ5D_index | Mean (SD) | 0.95 (0.1) | 0.85 (0.2) | <0.001 | 0.95 (0.1) | 0.86 (0.1) | 0.011 | |  |

Table S8 Overall Demographics – Per Protocol Dataset

This table summarizes the baseline demographic and clinical characteristics of participants included in the per protocol dataset. The table includes age, sex distribution, BMI, and EQ5D scores (VAS, Index, and dimensions). Statistical comparisons between SARS-CoV-2 positive and negative groups were conducted using similar methods to Table 14.

|  | | **Baseline** | | | **12 months** | | |
| --- | --- | --- | --- | --- | --- | --- | --- |
| **label** | **levels** | **SARSCoV2-Neg** | **SARSCoV2-Pos** | **P** | **SARSCoV2-Neg** | **SARSCoV2-Pos** | **P** |
| Age | Mean (SD) | 48.2 (7.3) | 48.5 (7.1) | 0.698 |  |  |  |
| Sex | Female | 252 (82.4) | 36 (40.0) | **<0.001** |  |  |  |
|  | Male | 54 (17.6) | 54 (60.0) |  |  |  |  |
| BMI | Mean (SD) | 26.6 (4.9) | 27.9 (3.7) | 0.014 |  |  |  |
| Mobility | No problems | 293 (95.8) | 72 (80.0) | **<0.001** | 290 (94.8) | 88 (97.8) | 0.076 |
|  | Some Problems | 12 (3.9) | 18 (20.0) |  | 12 (3.9) | 0 (0.0) |  |
|  | (Missing) | 1 (0.3) | 0 (0.0) |  | 4 (1.3) | 2 (2.2) |  |
| Selfcare | No problems | 300 (98.0) | 90 (100.0) | - | 282 (92.2) | 78 (86.7) | - |
|  | Some Problems | 0 (0.0) | 0 (0.0) |  | 0 (0.0) | 0 (0.0) |  |
|  | (Missing) | 6 (2.0) | 0 (0.0) |  | 24 (7.8) | 12 (13.3) |  |
| Activity | No problems | 293 (95.8) | 60 (66.7) | **<0.001** | 295 (96.4) | 76 (84.4) | **<0.001** |
|  | Some Problems | 12 (3.9) | 30 (33.3) |  | 7 (2.3) | 12 (13.3) |  |
|  | A lot of Problems | 0 (0.0) | 0 (0.0) |  | 0 (0.0) | 0 (0.0) |  |
|  | (Missing) | 1 (0.3) | 0 (0.0) |  | 4 (1.3) | 2 (2.2) |  |
| Pain | No problems | 299 (97.7) | 66 (73.3) | **<0.001** | 256 (83.7) | 68 (75.6) | 0.059 |
|  | Some Problems | 0 (0.0) | 24 (26.7) |  | 40 (13.1) | 20 (22.2) |  |
|  | A lot of Problems | 6 (2.0) | 0 (0.0) |  | 6 (2.0) | 0 (0.0) |  |
|  | (Missing) | 1 (0.3) | 0 (0.0) |  | 4 (1.3) | 2 (2.2) |  |
| Anxiety | No problems | 293 (95.8) | 54 (60.0) | **<0.001** | 266 (86.9) | 74 (82.2) | 0.154 |
|  | Some Problems | 12 (3.9) | 36 (40.0) |  | 30 (9.8) | 14 (15.6) |  |
|  | A lot of Problems | 0 (0.0) | 0 (0.0) |  | 6 (2.0) | 0 (0.0) |  |
|  | (Missing) | 1 (0.3) | 0 (0.0) |  | 4 (1.3) | 2 (2.2) |  |
| EQ5D_VAS | Mean (SD) | 86.8 (10.3) | 75.0 (14.1) | **<0.001** | 84.7 (12.1) | 76.2 (13.0) | **<0.001** |
| EQ5D_index | Mean (SD) | 0.9 (0.1) | 0.9 (0.1) | **<0.001** | 0.9 (0.1) | 0.9 (0.1) | 0.414 |

Table S9 Adjusted Analyses for EQ5D VAS and EQ5D Index at 12 Months

This table shows the adjusted regression analyses evaluating the association between SARS-CoV-2 status and EQ5D outcomes (VAS and Index) at 12 months. Models adjust for age, sex, BMI, and baseline scores (where applicable). Results are reported as adjusted estimates with standard errors (SE) and p-values. Separate analyses were conducted for the full dataset and per protocol dataset.

| **12 months** | **Full Dataset** | | **Per Protocol** | |
| --- | --- | --- | --- | --- |
|  | **Estimate (SE)** | **P value** | **Estimate (SE)** | **P value** |
| EQ5D-VAS adjusted for Age and BMI | -6.39 (1.35) | < 0.001 | -7.9 (1.5) | < 0.001 |
| EQ5D-Index adjusted for Age and BMI | -0.02 (0.01) | 0.049 | 0 (0.01) | 0.605 |
| EQ5D-VAS adjusted for Age, BMI and Baseline EQ5D- VAS | 1.24 (1.18) | 0.293 | -0.9 (1.37) | 0.511 |
| EQ5D-Index adjusted for Age, BMI and Baseline EQ5D-Index | 0.03 (0.01) | 0.003 | 0.04 (0.01) | < 0.001 |

Table S10 Likelihood of reporting problems in EQ5DL Dimensions at baseline and at 12 months.

This table provides results from logistic regression models assessing the likelihood of reporting problems in EQ5DL dimensions at 12 months. Adjusted estimates (standard error) and p-values are shown for each EQ5DL dimension, adjusted for covariates including age, sex, and BMI. Results are presented for baseline and 12 months for both datasets separately.

|  | **Full Dataset** | | | | **Per Protocol** | | | |
| --- | --- | --- | --- | --- | --- | --- | --- | --- |
| **EQ5DL Dimension** | **Baseline** | | **12 Months** | | **Baseline** | | **12 Months** | |
|  | **Adj. Estimate (SE)** | **P** | **Adj. Estimate (SE)** | **P** | **Adj. Estimate (SE)** | **P** | **Adj. Estimate (SE)** | **P** |
| Activity | 1.94 (0.29) | <0.001 | 0.69 (0.25) | 0.005 | 2.48 (0.37) | <0.001 | 2.05 (0.52) | <0.001 |
| Anxiety | 2.58 (0.33) | <0.001 | 0.19 (0.21) | 0.376 | 2.84 (0.38) | <0.001 | 0.23 (0.35) | 0.523 |
| Mobility | 1.38 (0.29) | <0.001 | 0.92 (0.28) | <0.001 | 1.79 (0.41) | <0.001 | -17.45 (1 867.32) | 0.993 |
| Pain | 3.33 (0.44) | <0.001 | 0.46 (0.2) | 0.025 | 2.94 (0.49) | <0.001 | 0.41 (0.31) | 0.188 |

Table S11 Changes in EQ5DL Dimensions from Baseline to 12 Months

This table reports the changes in EQ5DL dimensions (mobility, self-care, activity, pain, and anxiety) between baseline and 12 months for SARS-CoV-2 positive and negative groups. Results are stratified by dataset type (full vs. per protocol). P-values reflect the significance of group differences in change scores, adjusted for age, sex, and BMI.

| **EQ5DL Dimension** | **Full Dataset** | | **Per Protocol** | |
| --- | --- | --- | --- | --- |
|  | **Adj. Estimate (SE)** | **P** | **Adj. Estimate (SE)** | **P** |
| Activity | -0.128 (0.076) | 0.095 | -0.18 (0.027) | <0.001 |
| Anxiety | -0.262 (0.092) | 0.005 | -0.347 (0.121) | 0.006 |
| Mobility | -0.034 (0.066) | 0.609 | -0.2 (0.052) | 0.992 |
| Pain | -0.203 (0.088) | 0.024 | -0.168 (0.103) | 0.11 |

**Supplementary Figure 1 ABPM DBP Paired**


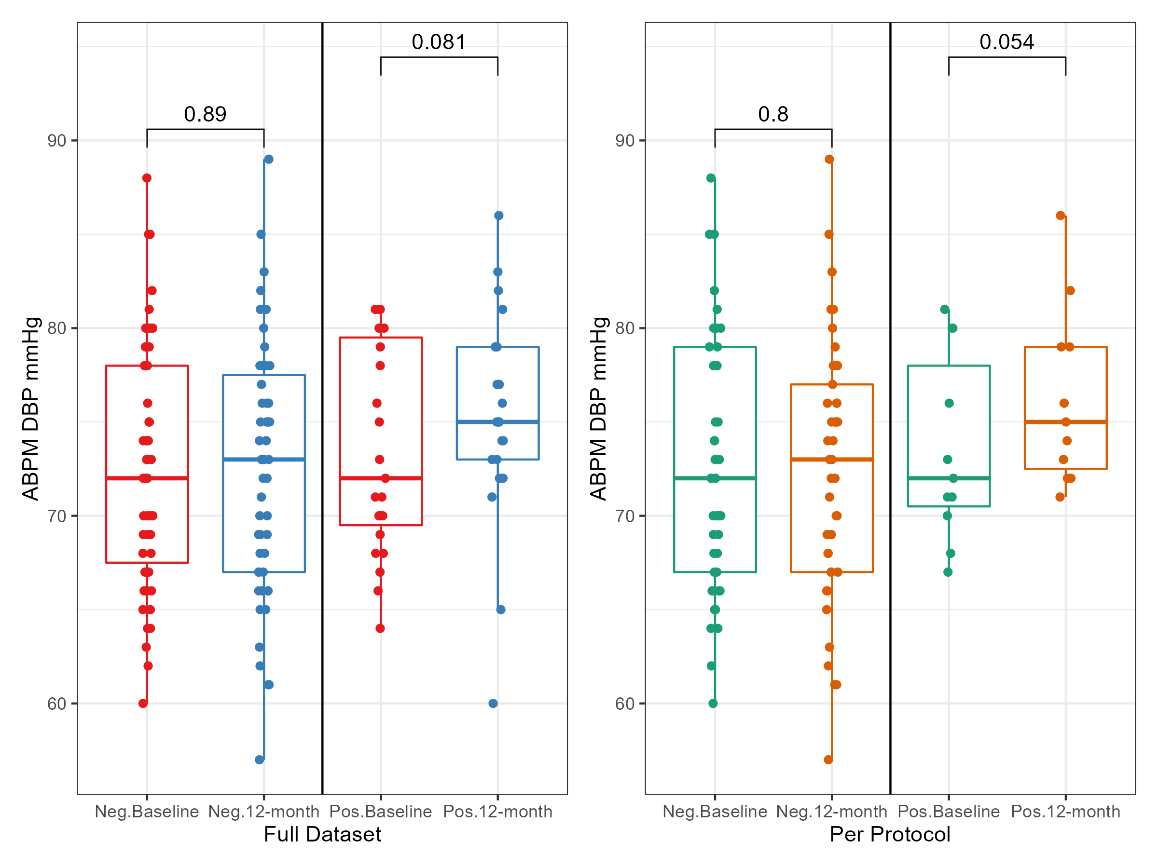


This figure illustrates the paired ambulatory blood pressure monitoring (ABPM) diastolic blood pressure (DBP) for the full dataset (left) and the per-protocol dataset (right). Each graph includes two panels: the left panel displays box plots for the SARS-CoV-2 negative group at baseline and 12 months, while the right panel shows box plots for the SARS-CoV-2 positive group at the same time points. The paired t-test p-values are indicated above the square brackets in each graph.

Supplementary Figure 2 6-Minute Walk Test


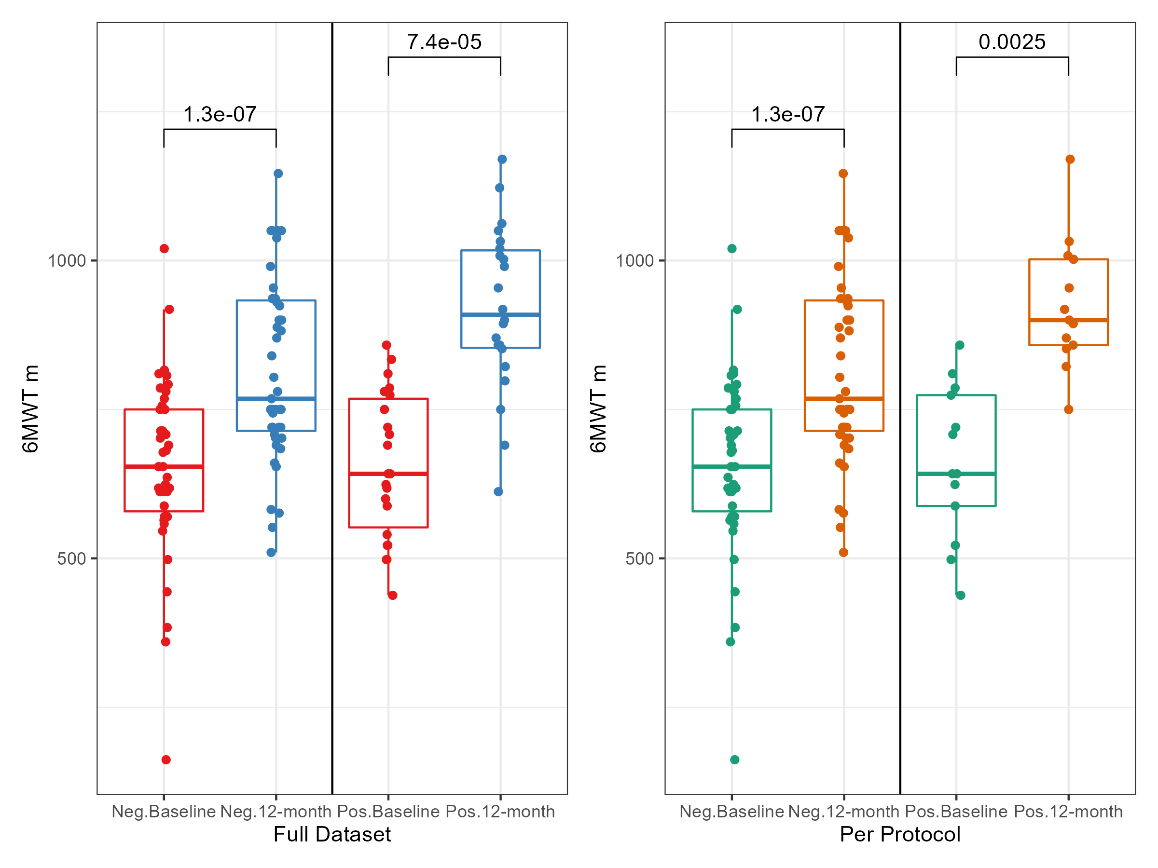


This figure illustrates the paired 6-MWT for the full dataset (left) and the per-protocol dataset (right). Each graph includes two panels: the left panel displays box plots for the SARS-CoV-2 negative group at baseline and 12 months, while the right panel shows box plots for the SARS-CoV-2 positive group at the same time points. The paired t-test p-values are indicated above the square brackets in each graph.

Supplementary Figure 3 Office SBP Paired


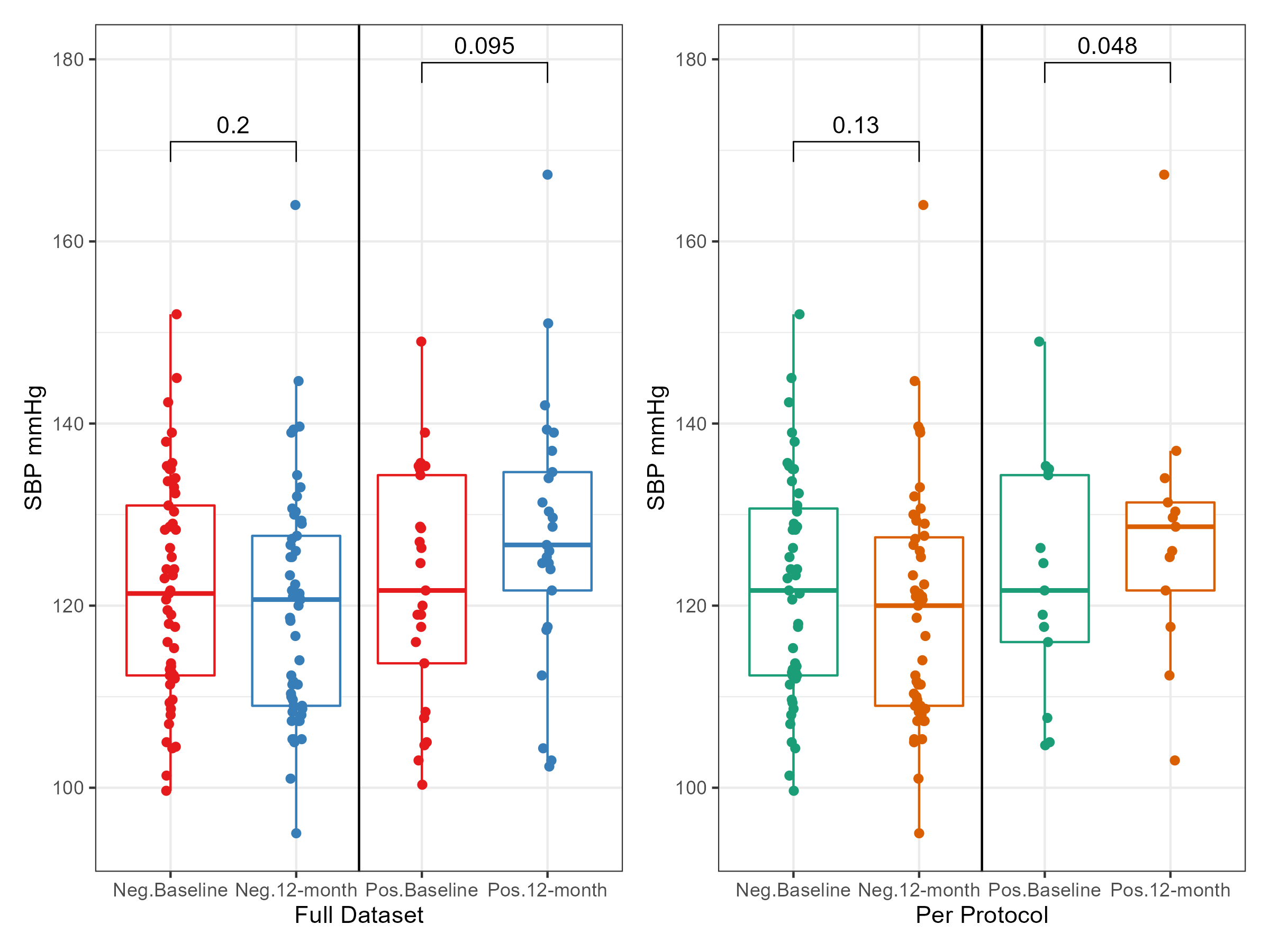


This figure illustrates the paired office SBP for the full dataset (left) and the per-protocol dataset (right). Each graph includes two panels: the left panel displays box plots for the SARS-CoV-2 negative group at baseline and 12 months, while the right panel shows box plots for the SARS-CoV-2 positive group at the same time points. The paired t-test p-values are indicated above the square brackets in each graph.

Supplementary Figure 4 Office DBP Paired


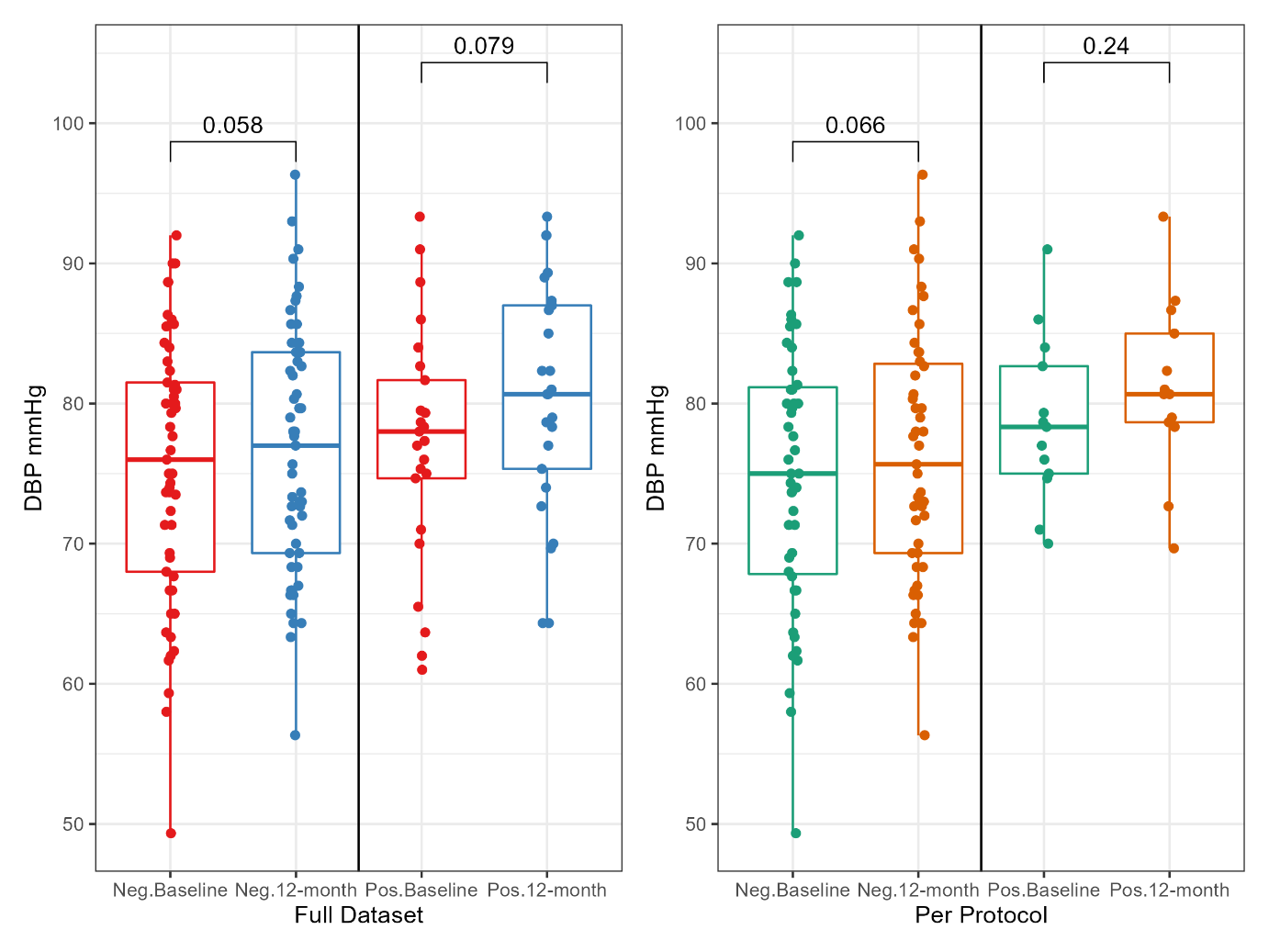


This figure illustrates the paired office DBP for the full dataset (left) and the per-protocol dataset (right). Each graph includes two panels: the left panel displays box plots for the SARS-CoV-2 negative group at baseline and 12 months, while the right panel shows box plots for the SARS-CoV-2 positive group at the same time points. The paired t-test p-values are indicated above the square brackets in each graph.

Supplementary Figure 5 Serum Sodium Paired


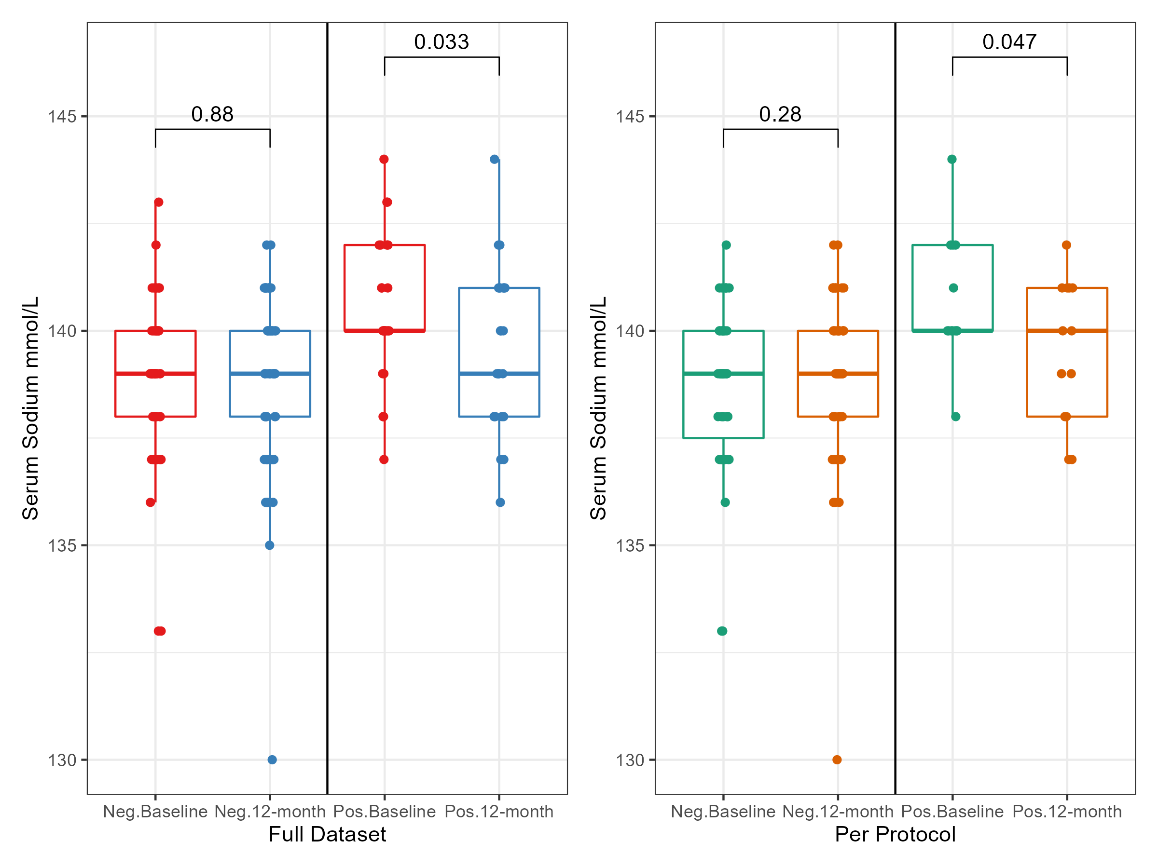


This figure illustrates the serum sodium for the full dataset (left) and the per-protocol dataset (right). Each graph includes two panels: the left panel displays box plots for the SARS-CoV-2 negative group at baseline and 12 months, while the right panel shows box plots for the SARS-CoV-2 positive group at the same time points. The paired t-test p-values are indicated above the square brackets in each graph.

Supplementary Figure 6 Serum HbA1c Paired


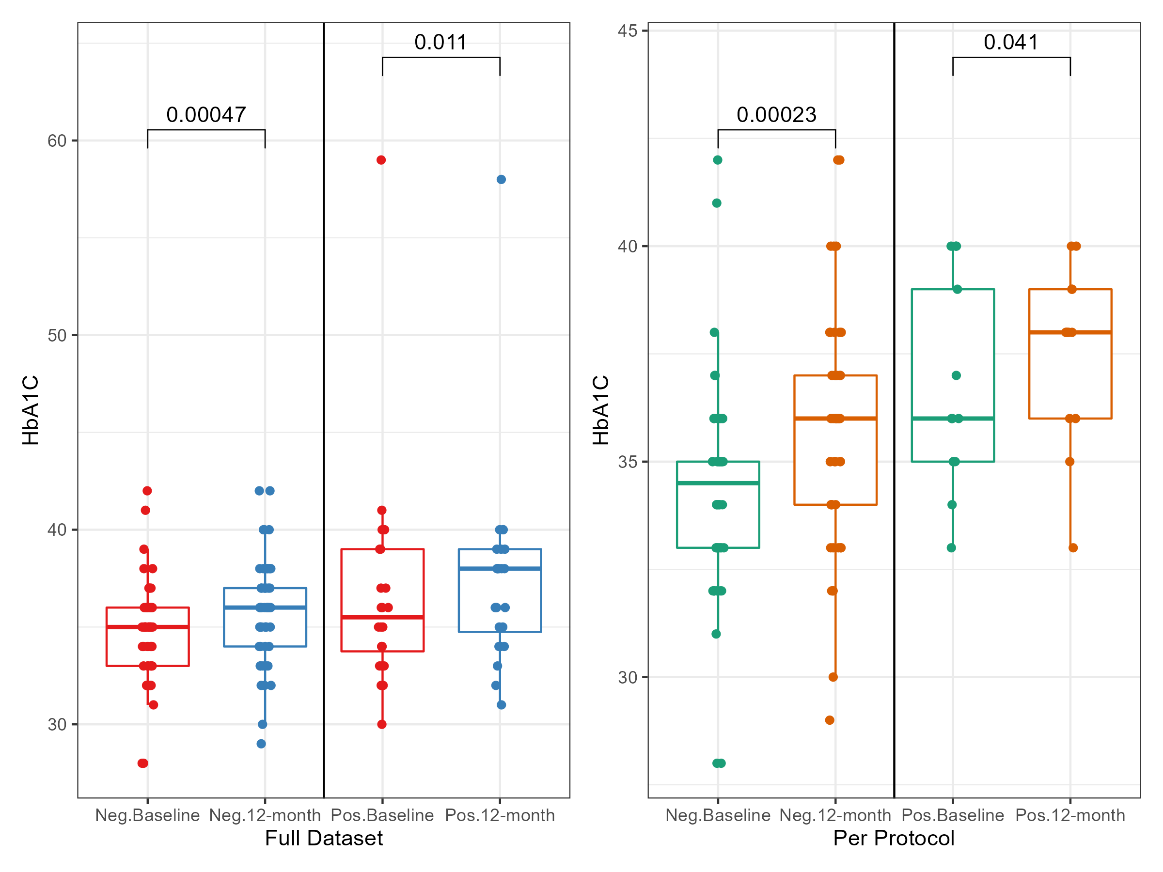


This figure illustrates the paired HbA1c for the full dataset (left) and the per-protocol dataset (right). Each graph includes two panels: the left panel displays box plots for the SARS-CoV-2 negative group at baseline and 12 months, while the right panel shows box plots for the SARS-CoV-2 positive group at the same time points. The paired t-test p-values are indicated above the square brackets in each graph.

Supplementary Figure 7 Serum Urea Paired


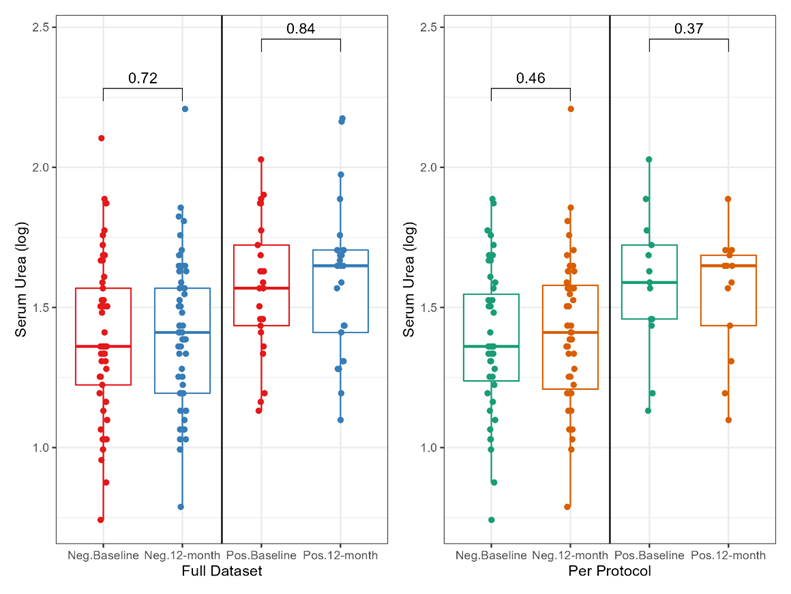


This figure illustrates the paired serum urea for the full dataset (left) and the per-protocol dataset (right). Each graph includes two panels: the left panel displays box plots for the SARS-CoV-2 negative group at baseline and 12 months, while the right panel shows box plots for the SARS-CoV-2 positive group at the same time points. The paired t-test p-values are indicated above the square brackets in each graph

**Supplementary Figure 8 Serum Creatinine**


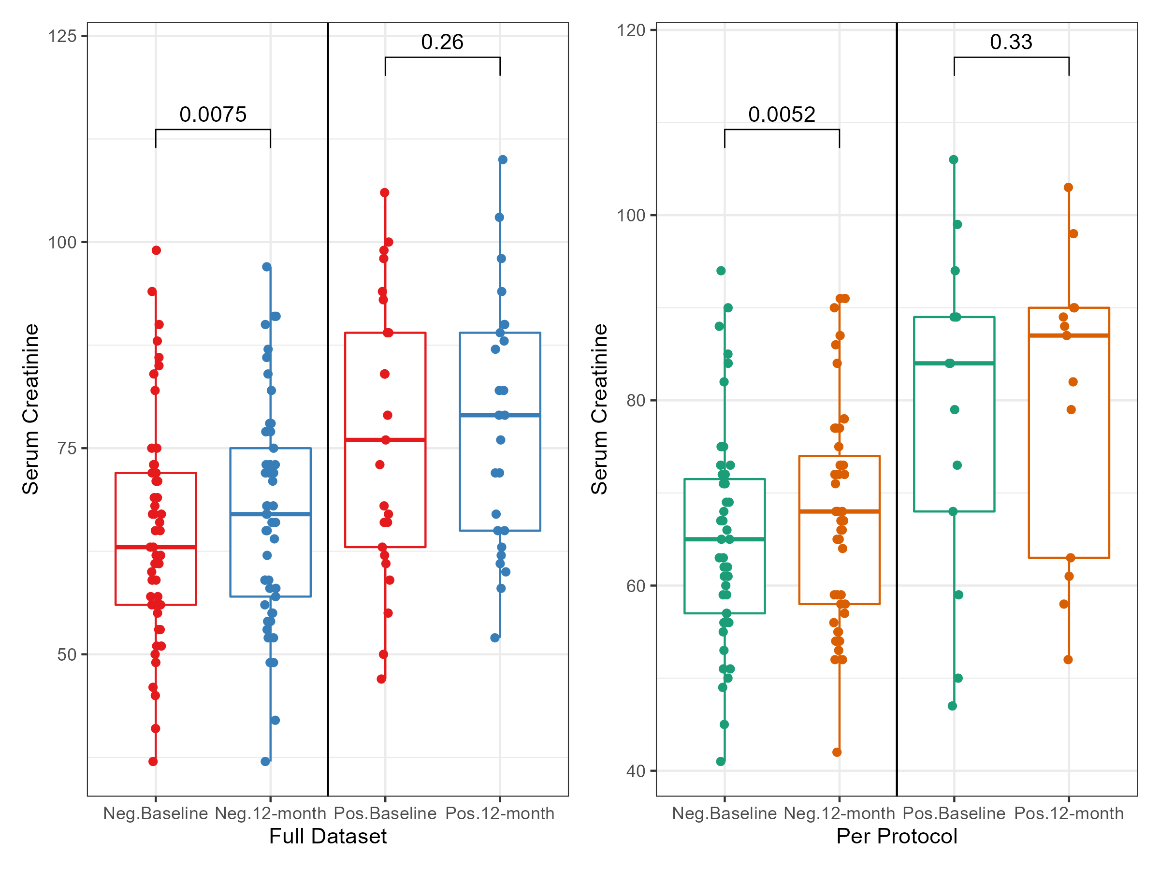


This figure illustrates the paired serum creatinine for the full dataset (left) and the per-protocol dataset (right). Each graph includes two panels: the left panel displays box plots for the SARS-CoV-2 negative group at baseline and 12 months, while the right panel shows box plots for the SARS-CoV-2 positive group at the same time points. The paired t-test p-values are indicated above the square brackets in each graph.

Supplementary Figure 9 Serum Haemoglobin (Hb) Paired


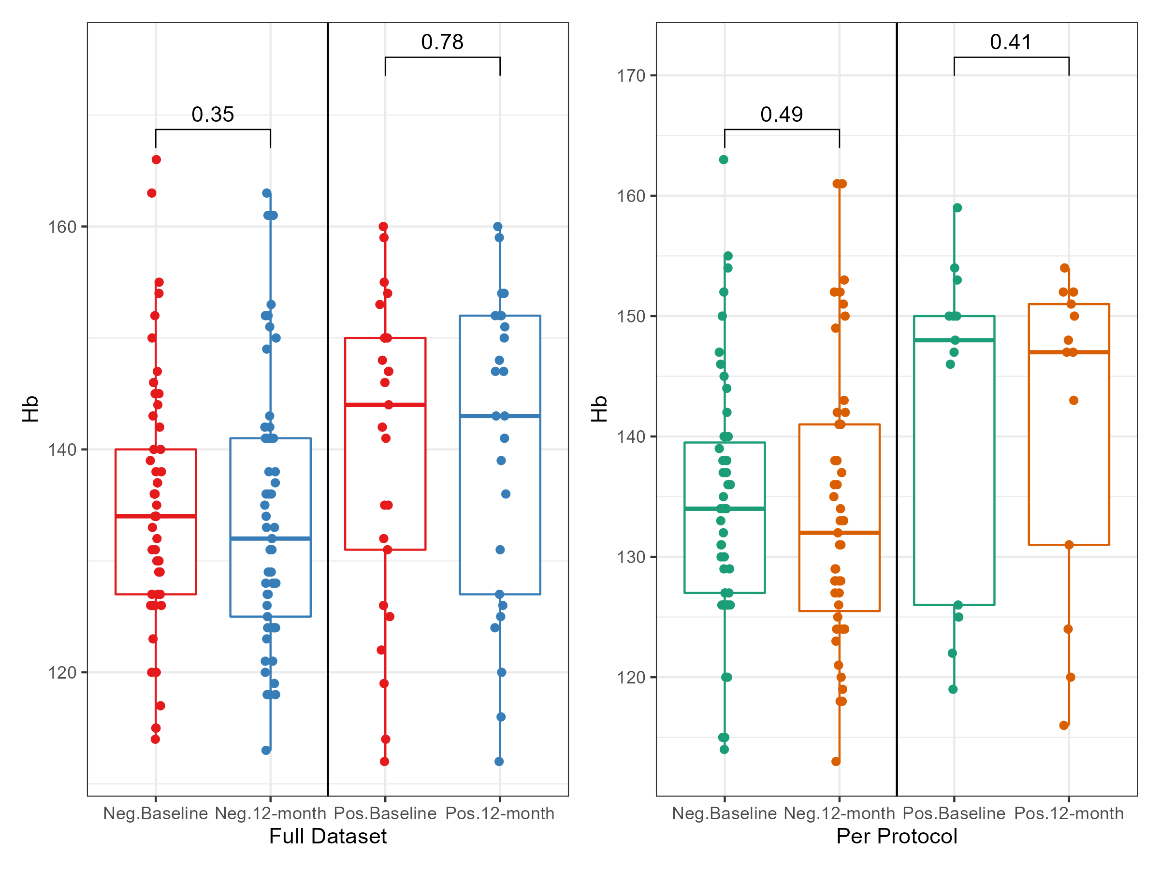


This figure illustrates the paired serum Hb for the full dataset (left) and the per-protocol dataset (right). Each graph includes two panels: the left panel displays box plots for the SARS-CoV-2 negative group at baseline and 12 months, while the right panel shows box plots for the SARS-CoV-2 positive group at the same time points. The paired t-test p-values are indicated above the square brackets in each graph.

**Supplementary Figure 10 Urine Sodium Creatinine ratio (uNaCr) Paired**


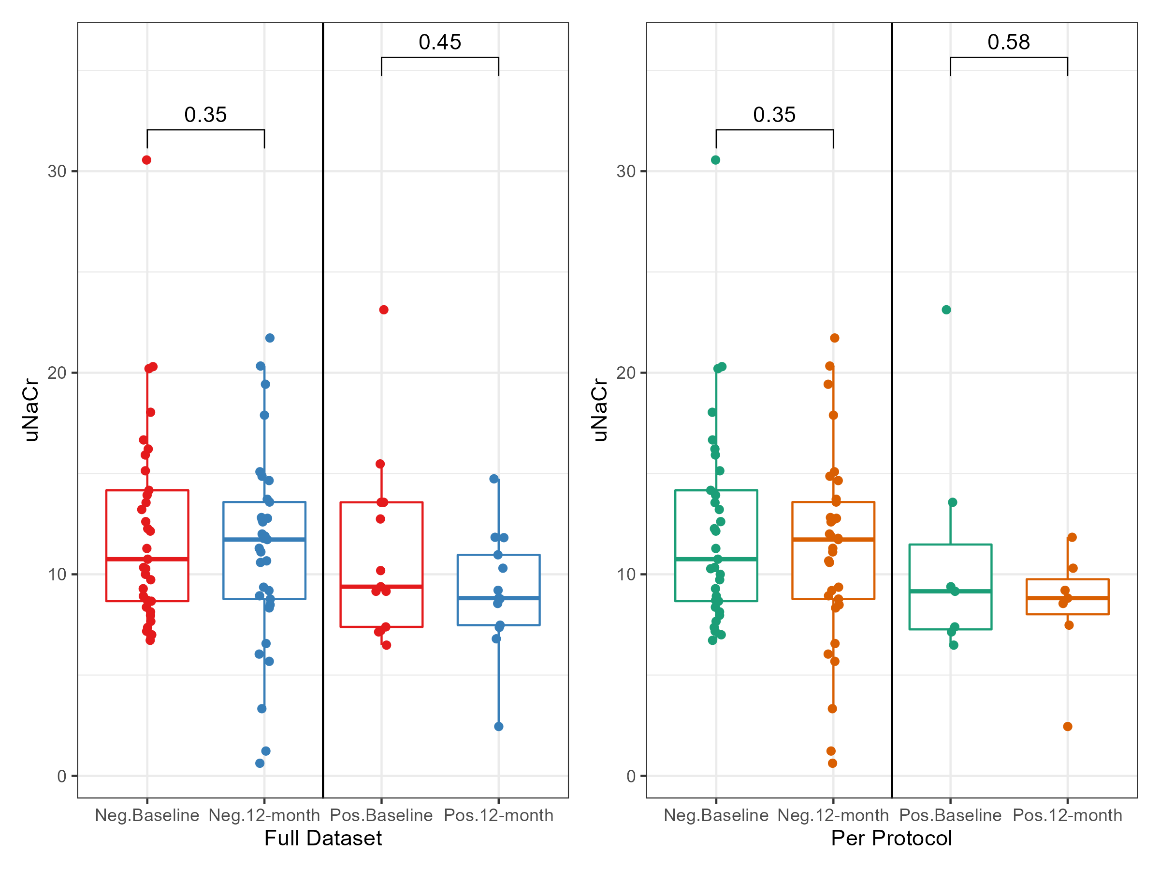


This figure illustrates the paired urine NaCr for the full dataset (left) and the per-protocol dataset (right). Each graph includes two panels: the left panel displays box plots for the SARS-CoV-2 negative group at baseline and 12 months, while the right panel shows box plots for the SARS-CoV-2 positive group at the same time points. The paired t-test p-values are indicated above the square brackets in each graph.

**Supplementary Figure** 11 **Urine Potassium Creatinine Ratio (uKCr) Paired**


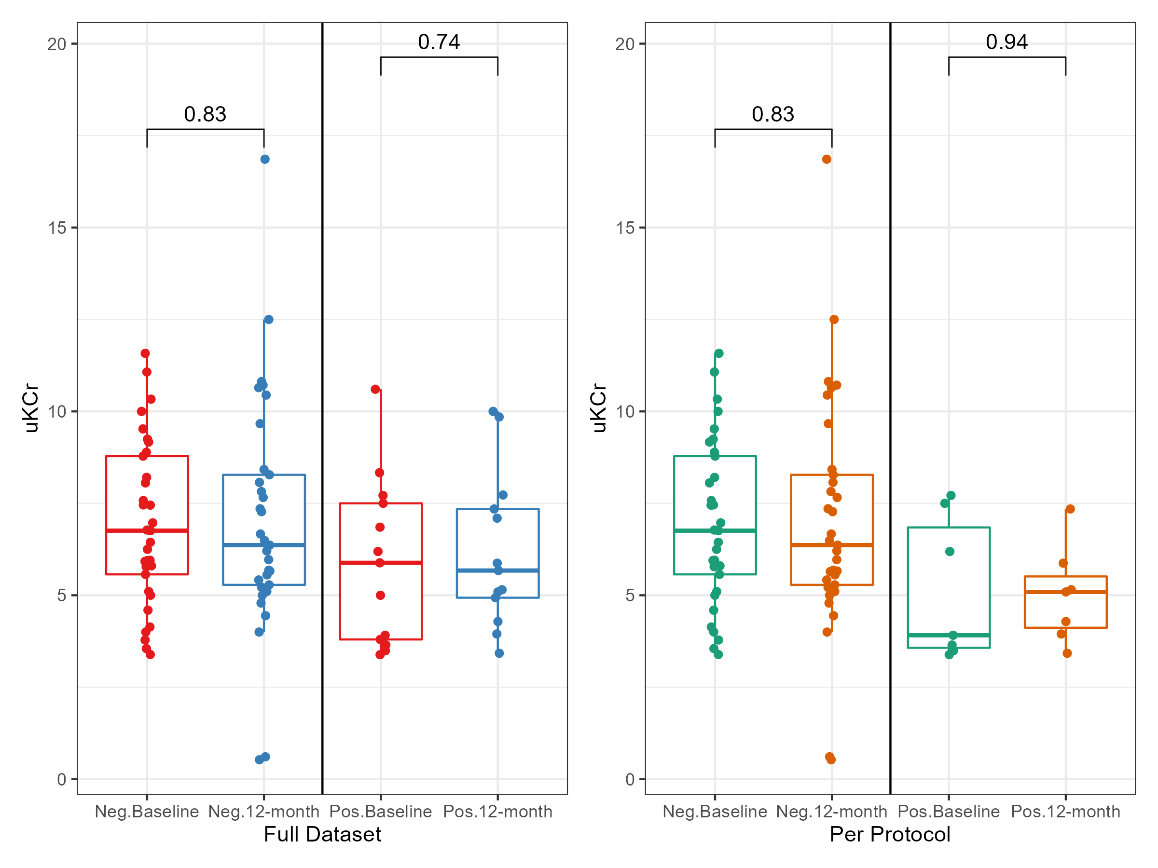


This figure illustrates the paired urine KCr for the full dataset (left) and the per-protocol dataset (right). Each graph includes two panels: the left panel displays box plots for the SARS-CoV-2 negative group at baseline and 12 months, while the right panel shows box plots for the SARS-CoV-2 positive group at the same time points. The paired t-test p-values are indicated above the square brackets in each graph.

Supplementary Figure 12 Changes in EQ5D-VAS Over Time

The box plot below shows the changes in EQ5D-VAS scores for SARS-CoV-2 positive (SARSCoV2-Pos) and negative (SARSCoV2-Neg) groups from baseline to 12 months for both the full dataset and per protocol dataset


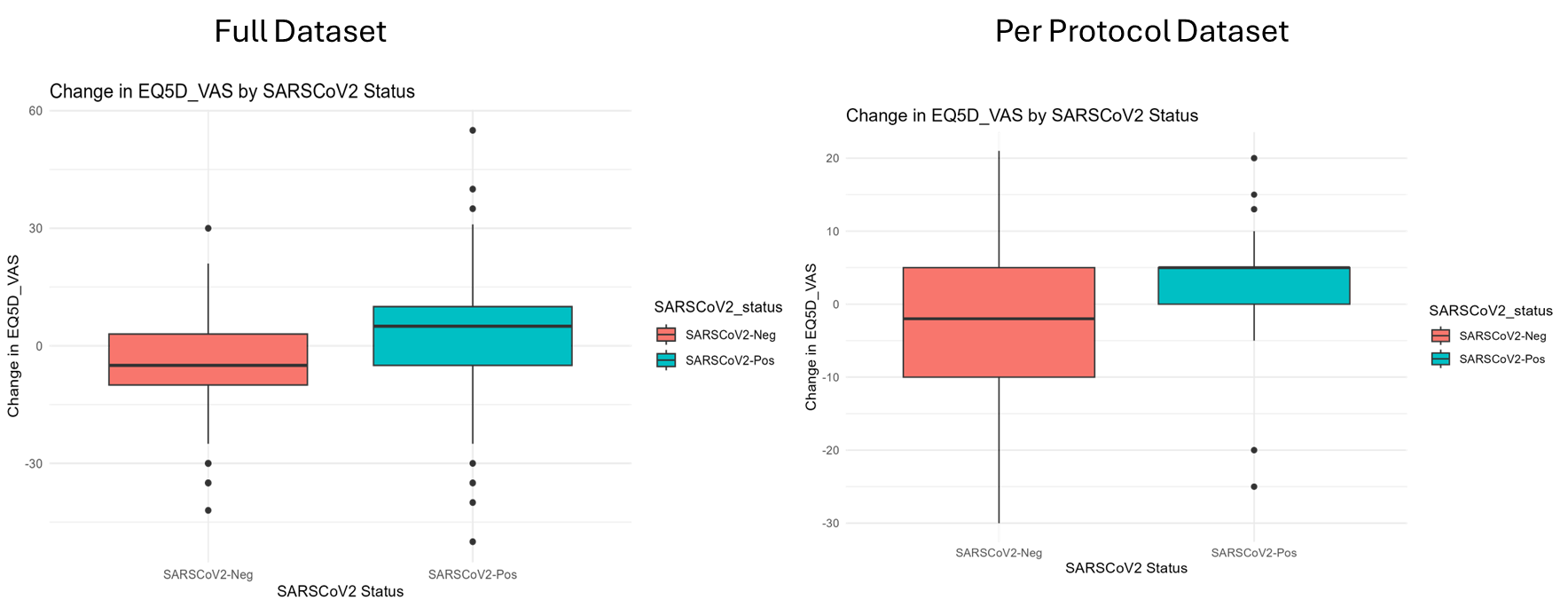


Supplementary Figure 13 Changes in EQ5D-Index Over Time

The box plot below shows the changes in EQ5D-Index scores for SARS-CoV-2 positive (SARSCoV2-Pos) and negative (SARSCoV2-Neg) groups from baseline to 12 months.


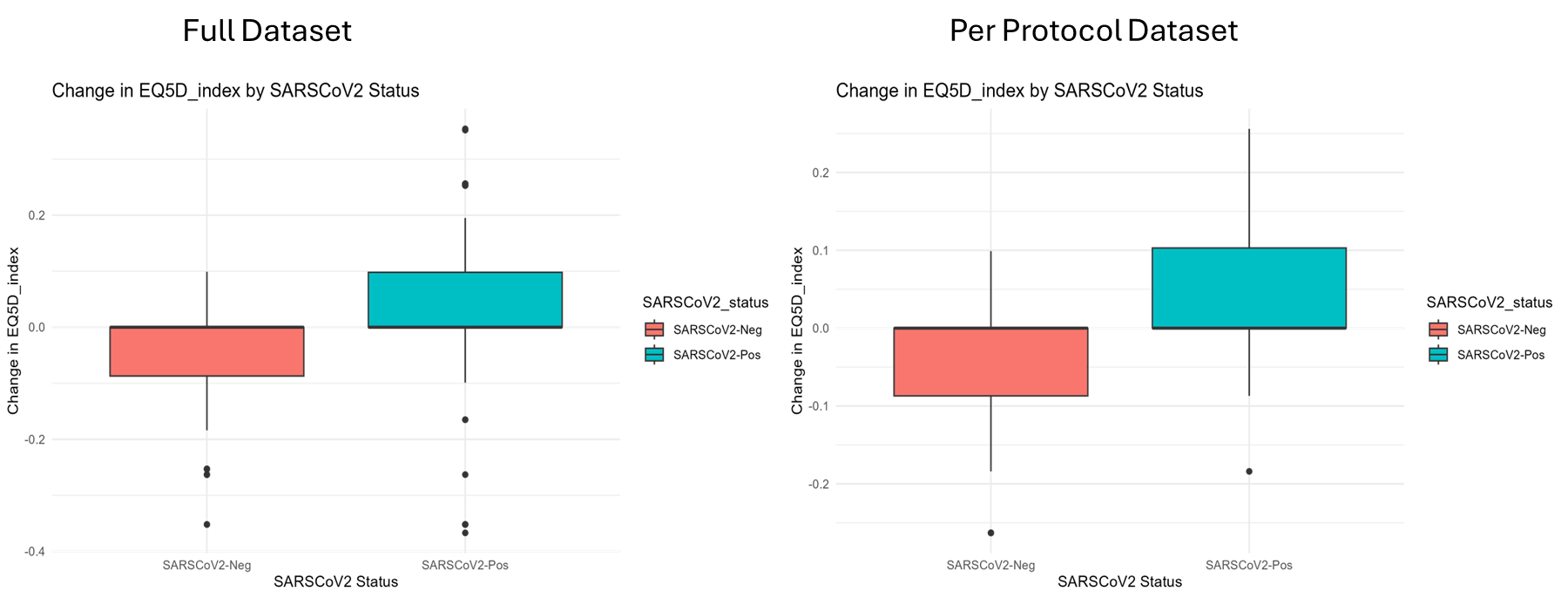

Supplement: Supplemental Digital Content [file jhype-43-1057-s001.docx]
